# CD177-mediated nanoparticle targeting of human and mouse neutrophils

**DOI:** 10.1371/journal.pone.0200444

**Published:** 2018-07-10

**Authors:** Heini M. Miettinen, Jeannie M. Gripentrog, Connie I. Lord, Jon O. Nagy

**Affiliations:** 1 Department of Microbiology and Immunology, Montana State University, Bozeman, MT, United States of America; 2 NanoValent Pharmaceuticals, Inc., Bozeman, MT, United States of America; Helsingin Yliopisto, FINLAND

## Abstract

Neutrophils are the most abundant white blood cells, with a vital role in innate immune defense against bacterial and fungal pathogens. Although mostly associated with pathological processes directly related to immune defense, they can also play a detrimental role in inflammatory conditions and have been found to have a pro-metastatic role in the spread of cancer cells. Here, we explore ways to temporarily suppress these detrimental activities. We first examined the possibility of using siRNA and antisense oligonucleotides (ASOs) for transient knockdown of the human and mouse C5a receptor, an important chemoattractant receptor involved in neutrophil-mediated injury that is associated with myocardial infarction, sepsis, and neurodegenerative diseases. We found that siRNAs and ASOs transfected into cultured cell lines can eliminate 70–90% of C5a receptor mRNA and protein within 72 h of administration, a clinically relevant time frame after a cardiovascular event. Targeted drug delivery to specific cells or tissues of interest in a mammalian host, however, remains a major challenge. Here, using phage display technology, we have identified peptides that bind specifically to CD177, a neutrophil-specific surface molecule. We have attached these peptides to fluorescent, lipid-based nanoparticles and confirmed targeting and delivery to cultured cells ectopically presenting either human or mouse CD177. In addition, we have shown peptide-nanoparticle binding specifically to neutrophils in human and mouse blood. We anticipate that these or related tagged nanoparticles may be therapeutically useful for delivery of siRNAs or ASOs to neutrophils for transient knockdown of pro-inflammatory proteins such as the C5a receptor.

## Introduction

Neutrophils (also known as polymorphonuclear leukocytes and neutrophilic granulocytes) are circulating innate immune cells that are recruited to sites of infection and injury. Upon arrival at these sites, they launch an inflammatory response that can result in further tissue damage and even death [1–5]. A major recruiter of neutrophils to such sites is C5a, a fragment of complement component C5, which is produced as a byproduct of the complement activation cascade triggered by cell and tissue damage [6]. C5a binds to a specific receptor (C5aR1) on circulating neutrophils; the quantity of C5aR1 increases upon inflammatory activation [7]. C5a is responsible for driving pathological inflammatory responses in a large number of diseases, such as ischemia reperfusion injury, neurodegenerative diseases, and sepsis [8–14]. Numerous experimental models have confirmed the importance of blocking the C5a-C5aR1 axis to limit the inflammatory damage caused by neutrophils at sites of tissue injury. For example, i) inhibition of complement activation before coronary occlusion reduced myocardial infarct size after reperfusion in rats [15]; ii) administration of anti-C5a antibody greatly improved rat survival in sepsis after cecal ligation and puncture, and significantly reduced myocardial neutrophil infiltration and coronary arteriolar endothelial injury in a porcine model of cardiopulmonary bypass and cardioplegic reperfusion [16, 17]; iii) antagonist inhibition of C5aR1 resulted in dramatic improvements in *in vivo* models of ischemia/reperfusion injury, sepsis, and septic cardiomyopathy [8, 9, 11, 13, 14, 18, 19]. Thus, the significance of C5a and C5aR1 in these disorders has been firmly established.

There are currently no effective drugs available for transient reduction of neutrophil damage caused by activation of the C5a-C5aR1 axis. Such a drug would be especially useful to reduce the severity of reperfusion injury. In the case of ischemic stroke, only 10–20% of patients undergo thrombolysis with tissue plasminogen activator (tPA) within the therapeutic time window of 3–4.5 hours [20]. tPA is currently the only FDA approved drug for ischemic stroke. Whether or not tPA is administered, suppression of inflammation is critical after an ischemic event to limit further tissue damage, such as prevention of cardiomyocyte death after a heart attack. In animal models, the blocking of C5aR1-mediated neutrophil activation has prevented not only severe pathology but also mortality [2, 3]. In this study, we examine C5aR1 knockdown using small inhibitory RNAs (siRNAs) and antisense oligonucelotides (ASOs). Since C5aR1 knockdown with siRNA and ASO can be expected to occur 4–72 h after administration, and neutrophil recruitment in stroke and myocardial infarction peaks at 24–48 h, it is anticipated that much of the damaging inflammatory effect caused by neutrophils could be ameliorated [21–23].

All current C5-C5aR drugs lack cell targeting specificity, leading to unwanted side effects. For example, C5aR1 modifies T-helper cell polarization and thus indirectly regulates the levels of IL-4 (a key regulator in humoral and adaptive immunity) and IFN-γ (critical for innate and adaptive immunity against viral and intracellular bacterial infections) [24]. C5aR1 is also found in hepatocytes, bronchial and alveolar cells, endothelial cells, astrocytes and microglia, with unknown effects upon blocking receptor function [25]. The following drugs against C5a or C5aR1 are either currently available, have been withdrawn, or are in preclinical trials: 1) Eculizumab (Soliris^®^) is a humanized antibody against C5, approved for treatment of rare disorders (paroxysmal nocturnal hemoglobinuria and atypical hemolytic uremic syndrome). Treatment with Soliris^®^ has caused serious meningococcal infections and failed in rheumatoid arthritis trials; 2) The previously FDA approved PMX-53, a C5aR antagonist, was discontinued in 2012 due to off-target side-effects [26–28]; 3) Avacopan (CCX-168) is a C5aR antagonist that is currently in phase III clinical studies for ANCA associated vasculitis [29]; 4) DF2593A is a C5aR negative allosteric modulator in preclinical studies against inflammatory neuropathic pain [30]. These therapeutics have critical limitations for use in humans and can adversely affect critical defense mechanisms against pathogens. A therapeutic agent that will specifically target the blood neutrophils and block their migration to sites of injury remains to be developed. Here, we examined the effect of siRNAs and synthetic antisense oligonucleotides (ASOs) on C5aR1 expression. One major advantage of using siRNA or ASOs is their transient nature, since a more prolonged knockdown of this important inflammatory receptor could be detrimental. The most challenging aspect of antisense therapeutics is efficient delivery of the siRNA or antisense oligonucleotide into the appropriate target cells. By selecting a surface receptor that is highly enriched on certain cell types, it is possible to minimize unwanted side effects. In the case of neutrophils, we narrowed the candidate receptor to one ideal receptor, CD177. CD177 (NB1, HNA-2a) is a glycosylphosphatidylinositol (GPI) anchored glycoprotein [31] expressed in blood exclusively by neutrophils, neutrophilic metamyelocytes, and myelocytes [32]. The precise function of CD177 has not been clarified, but it forms a complex with neutrophil proteinase 3 and binds to platelet endothelial cell adhesion molecule 1 (PECAM-1) [33, 34]. Crosslinking of CD177 does not activate inflammatory responses, such as degranulation and oxidative burst, but does induce internalization [35]. CD177 enhances neutrophil transmigration by binding PECAM-1 on endothelial cells, and antibodies that block CD177 binding to PECAM-1 inhibit neutrophil transmigration [34, 36–38]. CD177 is found at detectable levels on only 30–70% of circulating neutrophils, but since this population of neutrophils appears to have an advantage in transendothelial migration, it is an excellent target for decreasing the accumulation of neutrophils at sites of tissue injury [37, 39]. An estimated 1–10% of humans are CD177^null^ due to a CD177 pseudogene, which would exclude this population from CD177-mediated neutrophil targeting [35, 40–42]. In the current study, we used a bacteriophage random dodecapeptide library to identify peptides that bind specifically to human and mouse CD177. These peptides were displayed on nanoparticles for targeting of CD177-expressing Chinese hamster ovary (CHO) cells, as well as human and mouse neutrophils.

## Materials and methods

### Plasmids and cell lines

The construction and characterization of Chinese hamster ovary (CHO) cells expressing human C5a receptor 1 (C5aR1) and human C5aR1-green fluorescent protein (GFP) have been described previously [43]. pCMV6 mouse C5aR1 was purchased from OriGene Technologies (catalog number MC208206; GenBank Accession NM_007577). The mouse C5aR1-GFP construct was created by deleting the C5aR1 stop codon and inserting the GFP-coding cDNA into the *Mlu* I-*Not* I restriction sites in the pCMV6 vector. Human CD177 cDNA in the pDNR-Dual plasmid (clone HsCD00000952; GenBank Accession AY888472) was obtained from the DNASU Plasmid Repository. The cDNA was cloned into the constitutive expression vectors pBGSA and pBZSA to allow for selection in CHO cells with G418 and Zeocin (or Bleocin), respectively [44]. Mouse (*Mus musculus)* CD177 cDNA was obtained from OriGene Technologies in the pCMV6-Kan/Neo plasmid (catalog number MC201519; GenBank Accession BC027283). Since antibodies against mouse CD177 were not available at the start of this project, two different tags were tested: A c-myc tag (EQKLISEEDL) placed either two amino acids downstream from the predicted signal peptide cleavage site, or 4 or 21 amino acids upstream from the predicted single transmembrane domain, resulted in misfolded proteins that were mostly retained in the endoplasmic reticulum, based on immunofluorescence microscopy. Similarly, an HA tag (YPYDVPDYA) two amino acids downstream from the predicted signal peptide cleavage site caused partial ER retention. An HA tag four amino acids upstream from the predicted single transmembrane domain was successfully expressed on the cell surface with no evidence of intracellular retention. CHO cells expressing HA-tagged formyl peptide receptor 1 (HA-FPR1) were used in the phage display peptide library screening to eliminate HA-binding peptides [45]. The CHO cell line was obtained in 1987 by HMM while working in the laboratory of Dr. Ira Mellman at Yale University. This cell line is not listed in the Database for Cross-Contaminated or Misidentified Cell Lines by the International Cell Line Authentication Committee (ICLAC).

### Antibodies and peptides

Mouse monoclonal anti-human CD177 antibody, clone MEM-166, catalog # 551899, was from BD Biosciences. Mouse monoclonal anti-HA-tag antibody, HA.11 clone 16B12, catalog # MMS-101R, was from BAbCO/Covance Research Products. Rabbit polyclonal anti-mouse CD177 antibody, clone 1171A, catalog # MAB8186, was from R&D Systems. Mouse monoclonal anti-human LAMP2 antibody, clone H4B4, catalog # sc-18822 was from Santa Cruz, Inc. Mouse monoclonal anti-hamster LAMP2 antibody (UH3) was a generous gift from Dr. Bruce L. Granger [44]. The antibodies against human and mouse CD177 and against the HA-tag showed no binding to wild-type CHO cells by immunofluorescence microscopy, validating their target specificity. The mouse anti-hamster LAMP2 antibody has been previously validated by immunocytochemistry and western blot analysis [44]. Secondary Alexa Fluor 488 goat anti-mouse monoclonal antibody, catalog # A-21121, and Alexa Fluor 488 goat anti-rabbit antibody, catalog # A-11008, were from Molecular Probes. The human CD177-binding peptide (Peptide H) and the scrambled control peptide were synthesized by GenScript, and the mouse CD177-binding peptide was synthesized by Peptide 2.0 (sequences are listed in List A in S1 File).

### siRNAs and antisense oligonucleotides

All siRNAs were purchased from Dharmacon (ThermoScientific): ON-TARGET plus non-targeting siRNA#1 (catalog # D-001810-01) was used as negative control siRNA. GFP Duplex I (catalog # P-002048-01) was used as positive control siRNA (ref# 4303). ON-TARGET plus human C5aR1 siRNA pool consisting of four different 19-mers (catalog #J-005442) was used for silencing of human C5aR1-GFP. Due to relatively poor results using pooled mouse C5aR1 siRNAs (catalog # J-043176), each mouse C5aR1 siRNA was also purchased and tested separately (-5, -6, -7, -8) with only -6 resulting in significant knockdown (S2 Fig and S3 Fig). Antisense mouse C5aR1 LNA™ GapmeR oligonucleotides with a phosphorothioate backbone were produced by Exiqon, Inc., with design based on proprietary software (Design ID # 580726–1 and 580726–2). An ASO produced by Integrated DNA Technologies to knockdown mouse C5aR1 had no effect and was used as the non-target control (NTC). The siRNA and ASO sequences can be found in List B and List C in S1 File.

### HPLN materials and other reagents

Hybrid Polymerized Liposomal Nanoparticles (HPLNs) were synthesized by NanoValent Pharmaceuticals, Inc. (Bozeman, MT). The components comprising the HPLN liposomes are hydrogenated soy L-α-phosphatidylcholine (“hydrogenated soy PC”), cholesterol and 1,2-distearoyl-*sn*-glycero-3-phosphoethanolamine-N-[methoxy(polyethylene glycol)-2000] (“m-Peg_2000_-DSPE”) (all from Avanti Polar Lipids, Alabaster, Alabama), N-(5’-hydroxy-3’-oxypentyl)-10-12-pentacosadiynamide (“h-Peg_1_-PCDA”), and N-(methoxy(polyethylene glycol)-2000)-10-12-pentacosadiynamide (“h-Peg_2000_-PCDA”).

### Phage display peptide library screening

Two libraries, PhD-7 and PhD-12 (New England Biolabs), were used to screen for peptides that bind human CD177. To eliminate peptides that bind to CHO cells, 1 x 10^9^ phage in PBS containing calcium and magnesium (PBS^++^), and supplemented with 0.5% bovine serum albumin (BSA), were incubated three rounds with wild-type CHO cells plated on 10 cm tissue culture plates, each for 1 h at room temperature on a rocker. Unbound phage were then incubated with CHO cells expressing human CD177 on a 10 cm tissue culture plate for 3 h at room temperature. Cells were rinsed ten times with PBS^++^ with 0.5% BSA and 0.1% Tween-20. Cell-bound phage were eluted with 0.1 M glycine-HCl, pH 2.2, for 10 min on ice with rocking and removed from the plate and neutralized with Tris-HCl, pH 8.8. To release internalized phage, cells were then incubated with 1% TritonX-100, 0.1 M glycine-HCl, pH 2.2, for 10 min on ice, removed from the plate and neutralized. Cell debris was removed by centrifugation. The eluted phage were amplified according to the instructions by New England Biolabs and the peptide library screening was repeated two times as above. The library screening for mouse CD177-binding peptides was carried out as above, except CHO cells expressing HA-FPR were used instead of wild-type CHO cells to eliminate peptides that bound to the HA-tag (in addition to endogenous CHO molecules). The DNA sequences of the phage peptides bound and internalized by CHO human CD177 cells and by CHO mouse CD177-HA cells were determined after the third round of selection. The best peptide for each CD177 was selected by comparing binding and internalization by CHO cells expressing CD177 to cells without CD177.

### Synthesis of CD177-binding lipid (peptide H- or M-Peg_2000_-DSPE) and scrambled peptide lipid

The CD177-binding dodecapeptide lipid was prepared by first exposing 1.0 ml of an 8.8 mg/ml aqueous solution of cysteine-terminated dodecapeptides (Peptide H, Peptide M or scrambled) to resin-supported TCEP reducing agent (Sigma-Aldrich) by shaking for 15 minutes. The solution was filtered directly into a flask containing 17.6 mg of powdered 1, 2-distearoyl-*sn*-glycero-3-phosphoethanolamine-N-[maleimide(polyethylene glycol)-2000] (“mal-Peg_2000_-DSPE”) (Avanti Polar Lipids, Alabaster, Alabama) and stirred under nitrogen. The powdered DSPE lipid dissolved completely after several hours and was allowed to react overnight at ambient temperature. Thin layer chromatography (TLC) analysis on silica gel plates (Kieselgel 60 F_254_, Merck KGaA, Darmstadt, Germany) with chloroform/methanol solvent mixture (4/1) as eluent showed that all the mal-Peg_2000_-DSPE was consumed and converted to a lower Rf spot. The solution was dried to a waxy solid and redissolved in 1/1 chloroform/methanol and filtered through Celite yielding Peptide H, Peptide M or scrambled peptide-Peg_2000_-DSPE.

### Production of HPLN particles

Non-targetable HPLNs were prepared from h-PEG_1_PCDA, hydrogenated soy PC, cholesterol, m-Peg_2000_-DSPE and m-PEG_2000_-PCDA at a molar proportion of 14:43:32:10:1, and targetable HPLNs were prepared from h-PEG_1_PCDA, hydrogenated soy PC, cholesterol, Peptide H or Peptide M-Peg_2000_-DSPE and m-PEG_2000_-DSPE at a molar proportion of 14:43:32:10:1, according to the method previously described [46]. Briefly, lipids were mixed and evaporated *in vacuo* to a film. Deionized water was added to the films so as to give a 25 mM (total lipid and cholesterol) suspension. The suspension was heated via sonication at 70–80°C with a probe-tip sonicator (Fisher sonic dismembrator model 300) for 10 min. The resulting nearly clear solution was then cooled to 5°C for 12 h. After warming to ambient temperature, the liposomes were polymerized by UV light irradiation (254 nm) with a Spectrolinker XL-1000 UV Crosslinker (Spectronics Corp.) for 1 min. The resulting blue HPLNs were heated to 65°C for 5 min to convert them to the red (fluorescent) form. The colored solutions were syringe filtered through 0.2 μm cellulose acetate filters in order to remove trace insoluble contaminants. The HPLN concentration was determined using the ammonium ferrothiocyanate assay [47]. The particle size (average ~96 nm) was determined on Malvern ZetaSizer in water.

### Immunofluorescence microscopy

CHO cells stably expressing human CD177 or HA-tagged mouse CD177, grown on glass coverslips, were incubated with various HPLN nanoparticles at 75 μg/ml, as indicated in the Figure legends. Cells were washed to remove unbound HPLNs, fixed with 2.5% paraformaldehyde in PBS, permeabilized and blocked with 0.01% saponin/0.2% gelatin in PBS, and stained with primary and secondary fluorescent antibodies, as indicated in the Figure legends. In experiments examining internalization of HPLNs, cells were incubated with or without subtilisin (1 mg/ml) in PBS containing 1 mM DTT and 5 mM EDTA. Since this treatment resulted in loss of cell adherence, the cells were centrifuged at 300 rpm for 2 min onto glass slides using a Cytospin 4™ Cytocentrifuge. Human or mouse anticoagulated blood was incubated with HPLN nanoparticles at 50 μg/ml for the indicated times. Red blood cells were separated from buffy coat and plasma by 5 min centrifugation at 420 x g. Most of the plasma was discarded and the buffy coat was collected into a new tube. The remaining red blood cells in the buffy coat were lysed with ice cold water (3x), NaCl was added to a final concentration of 150 mM, and the neutrophils were pelleted and suspended in RPMI-1640 supplemented with 10% fetal bovine serum. The neutrophils were centrifuged at 300 rpm for 2 min onto glass slides using a Cytospin 4™ Cytocentrifuge. Cells were fixed 5 min in cold methanol, blocked in 0.2% gelatin/PBS and stained with primary and secondary fluorescent antibodies, as indicated in the Figure legends. Collection of human blood was approved by the Montana State University Institutional Review Board for the Protection of Human Subjects (FWA00000165 and Protocol Number: HM100909). Healthy volunteers gave written informed consent. Mouse blood was obtained from the Montana State University Animal Resources Center, a registered facility with the U.S. Department of Agriculture that complies with the Public Health Service Center Policy of Use of Laboratory Animals (AAALAC #713 and Animal Welfare #A3627-01.

### Flow cytometry

Human leukocytes were isolated from whole blood as described above. Cells were incubated on ice for 30 min in the presence or absence of mouse anti-human CD177 antibody, then centrifuged for 5 min at 420 x g and suspended in PBS supplemented with 10% fetal bovine serum. Cells were incubated for 30 min on ice in the presence or absence of Peptide H-HPLN (37.5 μg/ml) and Alexa Fluor 488 goat anti-mouse IgG (1:100). Leukocytes (and remaining red blood cells) were analyzed with a Becton-Dickinson Accuri C6 flow cytometer with a 488 nm excitation laser and 533/30 nm FL1 filter for detecting Alexa Fluor 488 and a 585/40 nm FL2 filter for detecting the HPLNs. The neutrophil population was gated in the FSC and SSC channels.

For siRNA studies, 5x10^4^ CHO cells expressing human C5aR1-GFP or mouse C5aR1-GFP were plated in 500 μl medium in 24-well plates one day prior to transfection. Cell were transfected with 25–100 nM siRNA using Dharmafect 1 (Dharmacon) or TransIT-X2 (Mirus Bio LLC) transfection reagents, according to the manufacturer’s protocol. For ASO studies, transfections were carried out with no ASO (mock transfected), 50 nM or 100 nM ASO (C5aR1 LNA GapmeR_1) or 100 nM non-targeting control (NTC) using Xfect™ RNA transfection reagent (Takara Bio USA) or TransIT®-Oligo transfection reagent (Mirus Bio, LLC) using the manufacturer’s protocol. Culture medium was replaced every 24 h. 72 h post transfection, cells were removed from tissue culture plates with trypsin-EDTA, centrifuged and suspended in PBS containing 10% FBS and 1 μg/ml propidium iodide (PI). Cell suspensions were analyzed with a Becton-Dickinson Accuri C6 flow cytometer with a 488 nm excitation laser and 533/30 nm FL1 filter for detecting GFP and a 585/40 nm FL2 filter for detecting PI fluorescence. Cells were gated in the FSC and SSC channels to exclude large/clumped cells and highly vacuolar cells (indicating poor health), and in the FL2 channel to exclude dead cells based on their PI fluorescence.

### Reverse transcription quantitative PCR (RT-qPCR)

Total RNA was purified 72 h post transfection from the same cells as had been analyzed in flow cytometry experiments using the Qiagen RNeasy Plus micro kit (catalog # 74034) and 100 ng total RNA was reverse transcribed using the Qiagen miScript II RT kit (catalog # 218160). qPCR was carried out in a Roche LightCycler® using 2x QuantiTect SYBR green mix (Qiagen; catalog # 204143), 200 nM of each primer, and cDNA equivalent of 5 ng reverse transcribed RNA. Primers for CHO housekeeping genes were selected from the study carried out by Bahr and coworkers [48]. Two different sets of mouse C5aR1 primers were used to control for possible annealing and experimental variability. Primer sequences are shown in List D in S1 File and cycling conditions are shown in Table A in S1 File. The data were analyzed using the ΔΔCq calculation using the technical notes provided by Dharmacon [49] based on the MIQE guidelines [50].

## Results and discussion

### Identification of peptides binding to mouse and human CD177

As outlined in the Introduction, one challenge in developing neutrophil-specific therapeutics is finding neutrophil-specific targets that do not activate those cells. We chose as our target CD177, a GPI-anchored glycoprotein with no known signaling function and limited expression in other cell types [31, 32]. Bacteriophage peptide libraries were used to select peptides that bind human and mouse CD177 stably expressed on the surfaces of CHO cells. Whereas selection with the PhD-7 library did not result in phage peptides with specific binding to human or mouse CD177, the PhD-12 library resulted in several promising dodecapeptides (Table 1). Further quantitative binding analysis identified the best peptides for mouse and human CD177 (Table 2). Neutrophil targeting molecules have previously been identified by two research groups: 1) Two human neutrophil-binding peptides were discovered using a random phage peptide display library [51], but the identities of the receptors remain unknown. The first peptide activated cell signaling through an unknown G protein coupled receptor and is therefore not ideal for therapeutic targeting because of this uncertainty [52]. The second peptide was found to target liposomal nanoparticles into monocytes as well as neutrophils, and therefore lacks cell type specificity [53]. 2) Wang *et al*. showed that albumin-coated nanoparticles could be targeted to activated neutrophils bound to vascular endothelial cells; these nanoparticles did not contain a targeting peptide, but relied on endocytosis mediated in part by the FcγIII receptor on the neutrophils and by other unknown receptor(s) [54, 55]. The advantages of our peptides over those listed above are three-fold: 1) the receptor is known; 2) the peptide binding does not cause neutrophil activation; 3) the receptor is neutrophil-specific.

**Table 1:** Amino acid sequences of peptides that bind human and mouse CD177.

**Human CD177-binding peptides**
I N H Q L D T T Q I L V (5)
F P L E T S H M S A P L (4)
Y S S A L K T L P I F Q (2)
K V F E Q D L L T T I L (2)
S M Q L M T S R L T W N (1)
N I L T T T W L P L H G (1)
T E L T T H P V F Q F R (1)
Y Q G D A N L K T W H V (1)
N L V T K P L H V S H L (1)
**Mouse CD177-binding peptides**
D F Y K P M P N L R I T (10)
W G F K P M D S L V I A (3)
D R W V A R D P A S I F (1)
S L D G A G A A L R T S (1)
G F S H S I P K L V I S (1)
S G S T P L F Q I F P Y (1)

The New England Biolabs PhD12 library was used to identify peptides that bind human and mouse CD177 expressed on CHO cells. The amino acid sequences obtained from the 3^rd^ round of library screening are shown. The number in parenthesis after the amino acid sequence indicates the number of times each sequence was obtained.

**Table 2:** Quantitative phage peptide binding assay.

**Human CD177-binding peptides**	**CHO Hu CD177 (pfu/ml)**	**CHO wild-type (pfu/ml)**	**Fold difference**
I N H Q L D T T Q I L V	1.1 x 10^9^	5.7 x 10^4^	1,930
F P L E T S H M S A P L	2.5 x 10^9^	1.8 x 10^4^	13,889
Y S S A L K T L P I F Q	2.1 x 10^9^	5.0 x 10^4^	4,200
K V F E Q D L L T T I L	1.7 x 10^9^	2.6 x 10^4^	6,538
S M Q L M T S R L T W N	5.8 x 10^9^	7.8 x 10^4^	7,436
N I L T T T W L P L H G	7.8 x 10^9^	32 x 10^4^	245
**Mouse CD177-binding peptides**	**CHO Ms CD177-HA (pfu/ml)**	**CHO HA-FPR (pfu/ml)**	**Fold difference**
D F Y K P M P N L R I T	1.6 x 10^9^	1.0 x 10^5^	15,800
W G F K P M D S L V I A	4.3 x 10^8^	1.1 x 10^5^	3,900

1 x 10^10^ phage were added to wild-type CHO cells, CHO human (Hu) CD177 cells, CHO HA-FPR cells or CHO mouse (Ms) CD177-HA cells. After 3 h incubation at room temperature, cells were washed extensively and the bound and internalized phage were eluted with 0.1 M glycine-HCl, pH 2.2, 1% Triton X-100. Titration was carried out using the manufacturer’s protocol. The titers shown for each phage peptide are shown as pfu/ml. The highlighted peptides were used in the subsequent experiments.

### Generation and testing of HPLNs with targeting peptides

Initially, the targeting peptides were expressed as genetic fusions on the coat protein of *Salmonella typhimurium* bacteriophage P22 viral-like particle and on the phage decoration protein, Dec [56, 57]. However, binding experiments with CHO cells expressing human or mouse CD177 gave poor and inconsistent results. Next, we explored HPLNs that have been previously used for cell targeting [58]. The peptides were synthesized with a glycine spacer and a C-terminal cysteine for chemical conjugation to mal-Peg_2000_-DSPE. These hybrid polymerized liposomal nanoparticles (HPLNs) share many structural attributes of conventional liposomes. They are self-assembling, unilamellar spheres whose surfaces can be modified using the same chemical coupling strategies as employed for liposomes. Unlike liposomes, HPLNs can be manufactured to be intrinsically fluorescent. Ultraviolet irradiation leads to cross-linking of diacetylene residues present in their acyl chains, leading to highly colored blue particles, and heat treatment of the HPLN vesicles leads to color change and fluorophore formation [59–61]. The fluorescence emission spectrum peaks at 550–570 nm with a broad and complex excitation spectrum from 470–540 nm. As a result, HPLNs converted into their fluorescent form can be readily traced from the time they bind to target cells until they are deposited and compartmentalized into subcellular structures.

**Fig 1:**
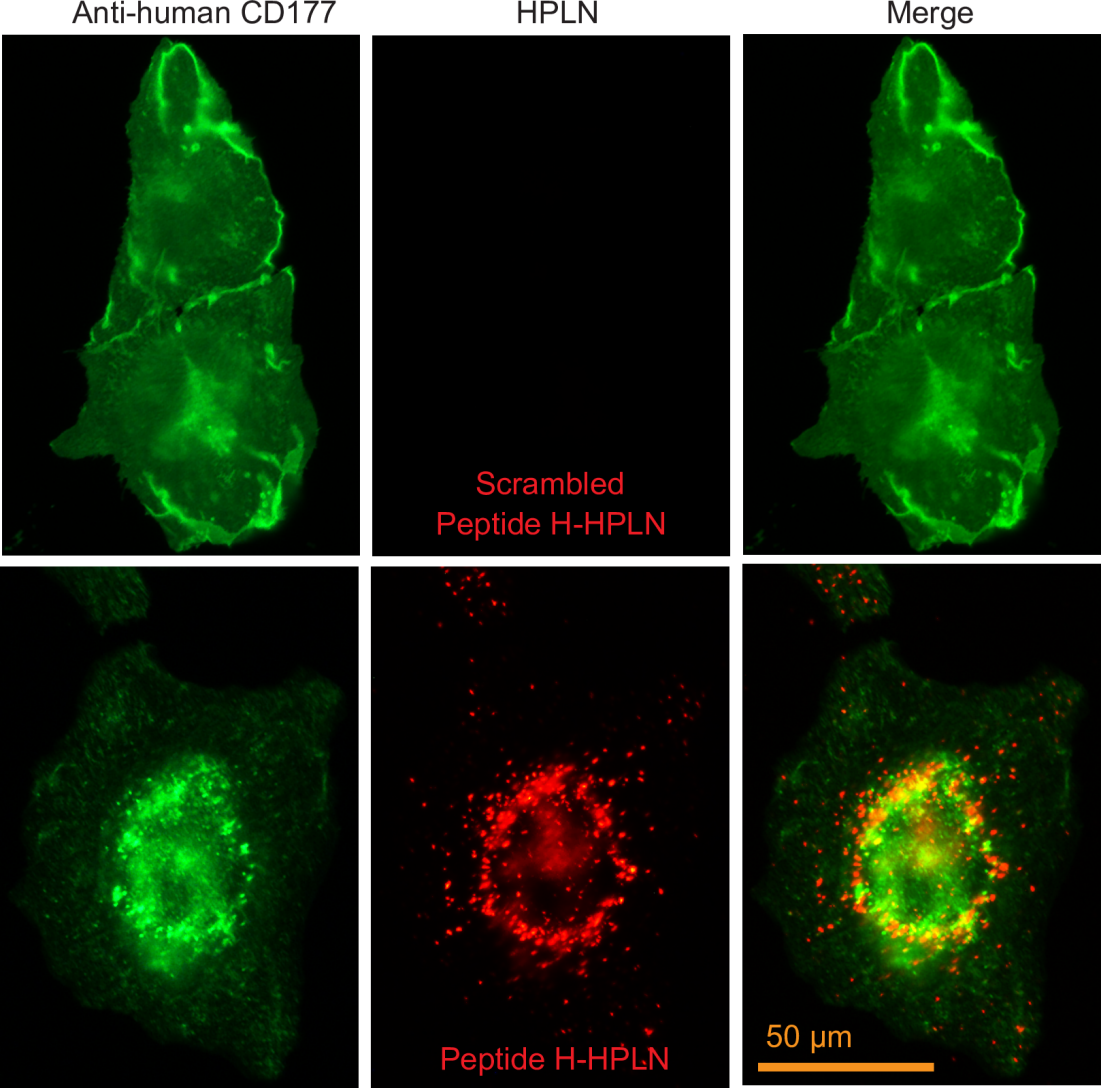
HPLNs displaying Peptide H bind human CD177 on cells. CHO cells expressing human CD177 were incubated for 3 h at 37°C with 75 μg/ml Peptide H-HPLNs. Cells were fixed and permeabilized, and CD177 was detected using a mouse anti-human CD177 antibody and an Alexa Fluor 488 goat anti-mouse secondary antibody. The HPLN particles are fluorescent red. As a negative control, HPLN particles displaying a scrambled peptide were used. The experiment was carried out twice, with at least 100 cells examined in each experiment. A direct correlation between the expression level of CD177 (based on fluorescence intensity) and the Peptide H-HPLN signal could be observed. The cell in the lower panel shows a typical cell distribution of CD177 and Peptide H-HPLNs after 3 h incubation. In contrast, the two cells shown in the upper panel are characteristic of the CD177 staining in the absence of Peptide H-HPLNs and in the presence of Scrambled Peptide H-HPLNs. Scale bar, 50 μm.

**Fig 2:**
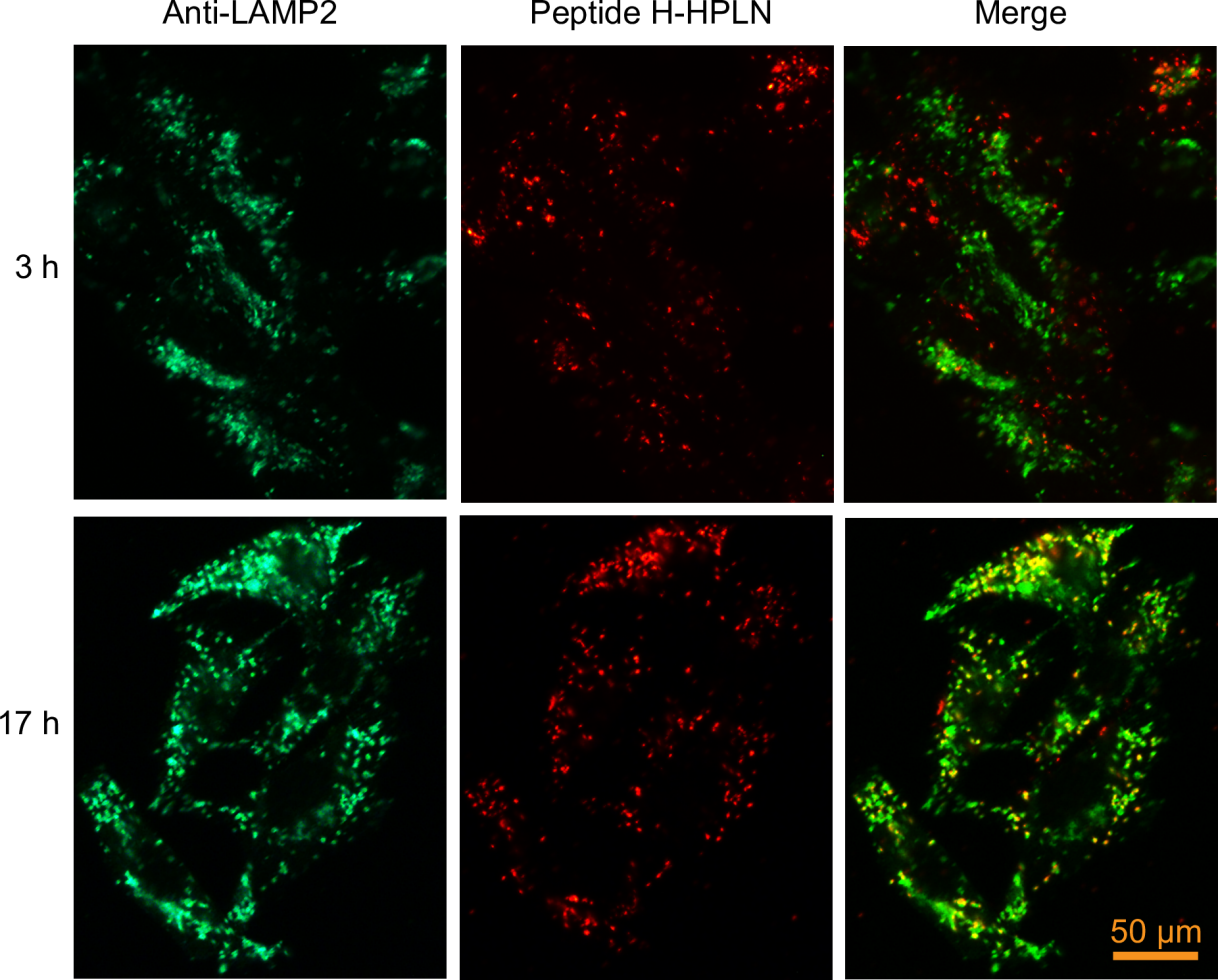
Some Peptide H-HPLN particles co-localize with LAMP2 after prolonged incubation, suggesting delivery into the lysosomes. CHO cells expressing human CD177 were incubated for 3 h and 17 h at 37°C with 75 μg/ml Peptide H-HPLN particles. Cells were fixed and permeabilized, and the lysosomes were stained with a mouse anti-hamster LAMP2 antibody and an Alexa Fluor 488 goat anti-mouse secondary antibody. The experiment was carried out once. The selected micrographs are representative of more than 100 cells with similar labeling patterns. Scale bar, 50 μm.

**Fig 3:**
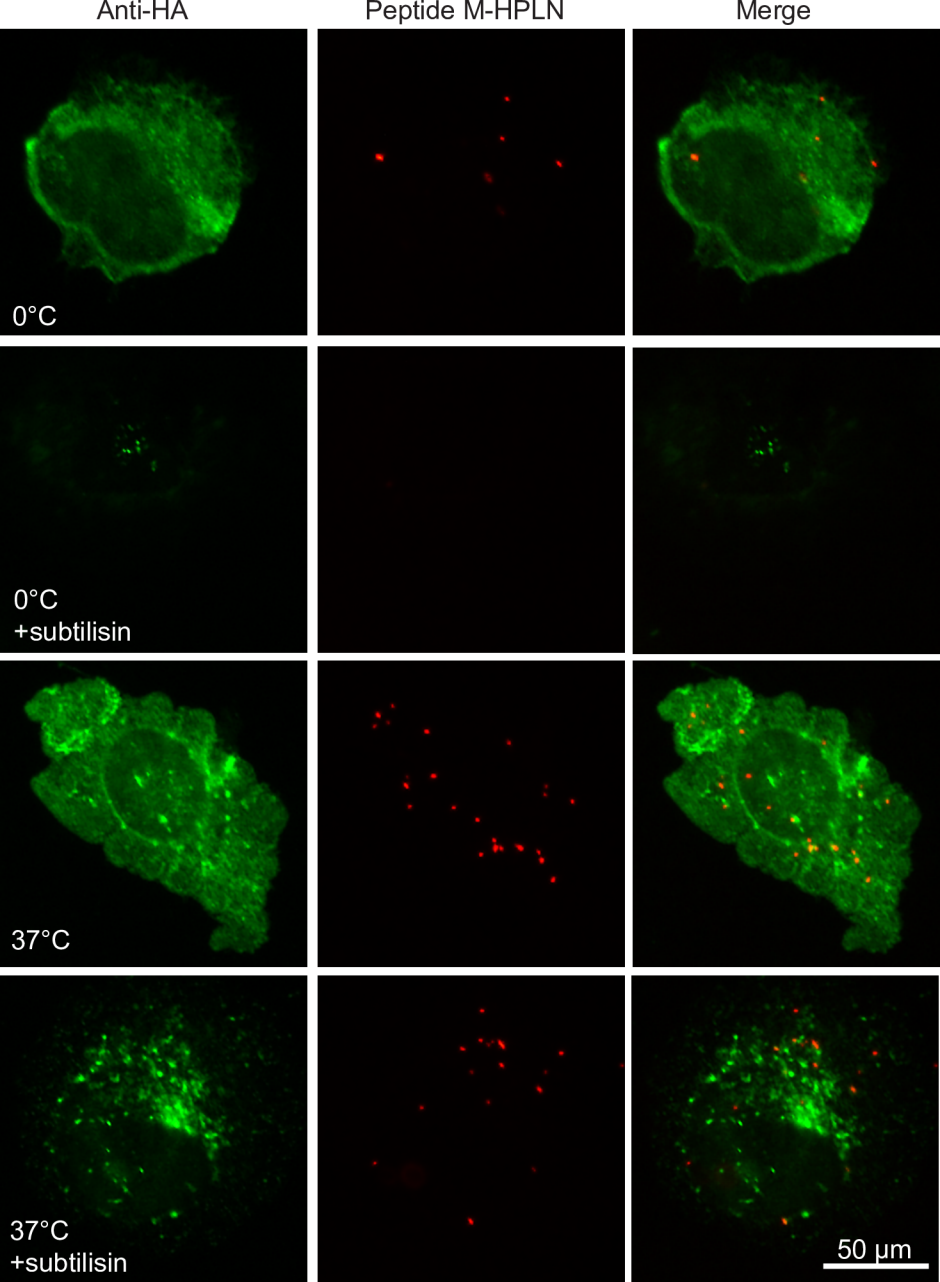
HPLN particles displaying Peptide M become internalized by CHO cells expressing mouse CD177-HA. CHO cells expressing mouse CD177 containing an HA-tag were incubated with 75 μg/ml Peptide M-HPLN particles for 2 h on ice to allow binding. Cells were then washed and kept on ice or warmed to 37°C for 1 h. Cells were then treated with subtilisin to remove surface-bound Peptide M-HPLN particles, or treated with buffer alone. After fixation and permeabilization, mouse CD177-HA was stained with a mouse anti-HA antibody and an Alexa Fluor 488 goat anti-mouse secondary antibody. The experiment was carried out once. Most cells out of more than 100 cells visualized on the slides had a similar staining pattern as those seen in these micrographs. Scale bar, 50 μm.

Binding and internalization was first examined using CHO cells stably expressing human CD177 or mouse CD177 with an HA-tag (CD177-HA). Incubation of the human peptide (Peptide H) HPLN particles with CHO human CD177 cells showed binding and apparent internalization of the HPLNs, whereas HPLN particles with a scrambled peptide showed no binding or internalization (Fig 1). Since delivery to lysosomes could result in nucleotide degradation by lysosomal enzymes, we examined the co-localization of the HPLN particles with the lysosomal marker, LAMP2. No co-localization was apparent up to 3 h of continuous incubation with the nanoparticles, but 17 h incubation resulted in some co-localization, suggesting that at least some of the nanoparticles are eventually routed to lysosomes (Fig 2). Similarly, HPLNs with the mouse CD177 binding peptide (Peptide M), were shown to bind CHO cells expressing mouse CD177-HA (Fig 3). To further confirm that the HPLN particles become internalized, we examined the distribution of the Peptide M-containing particles after treatment with subtilisin, a protease that will cleave HPLN particles from the cells unless endocytosis has taken place. Cells were incubated on ice with Peptide M-HPLN particles and either moved to 37°C to allow internalization, or kept on ice. Cells were then incubated with subtilisin, followed by fixation and processing for immunofluorescence microscopy. As shown in Fig 3, subtilisin treatment not only removed the surface bound HPLN particles, but also mouse CD177-HA, whereas internalized particles and mouse CD177-HA (37°C) were protected from protease activity.

### HPLNs show specificity for neutrophils when incubated with whole blood from humans and mice

**Fig 4:**
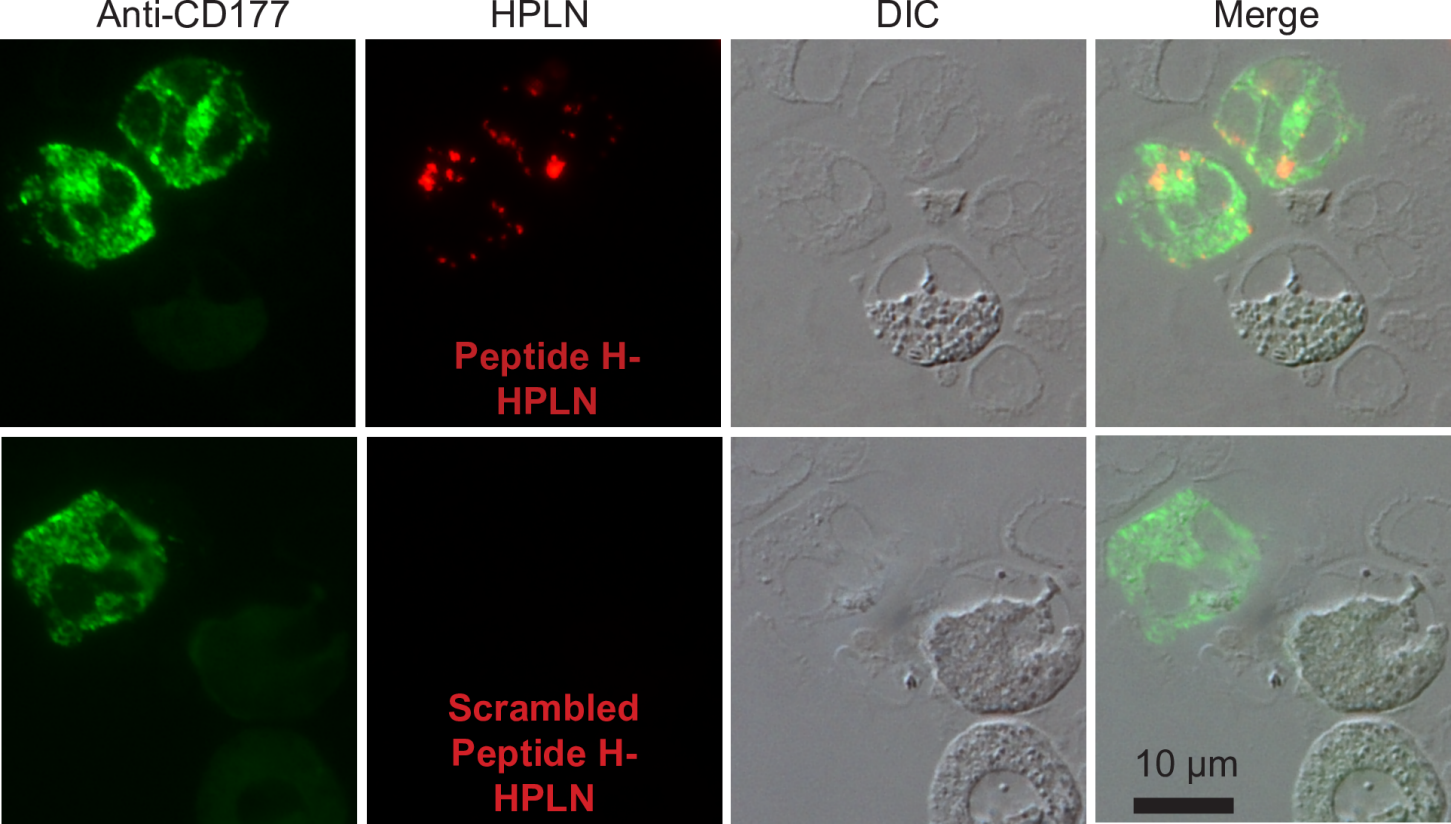
Peptide H-HPLN particles bind CD177-expressing human neutrophils in whole blood. Peptide H-HPLN particles were incubated with whole human blood for 1 h at 37°C. The white blood cells were collected from the buffy coat and the red blood cells were lysed. The white blood cells were centrifuged onto glass slides, fixed in methanol and stained with mouse anti-human CD177 antibody and an Alexa Fluor 488 goat anti-mouse secondary antibody. Scrambled Peptide H-HPLN particles were used as a negative control. The experiment was carried out four times using blood from four different donors. In each case, slides with several hundred cells were examined and the micrographs are representative of these results. Scale bar, 10 μm.

**Fig 5:**
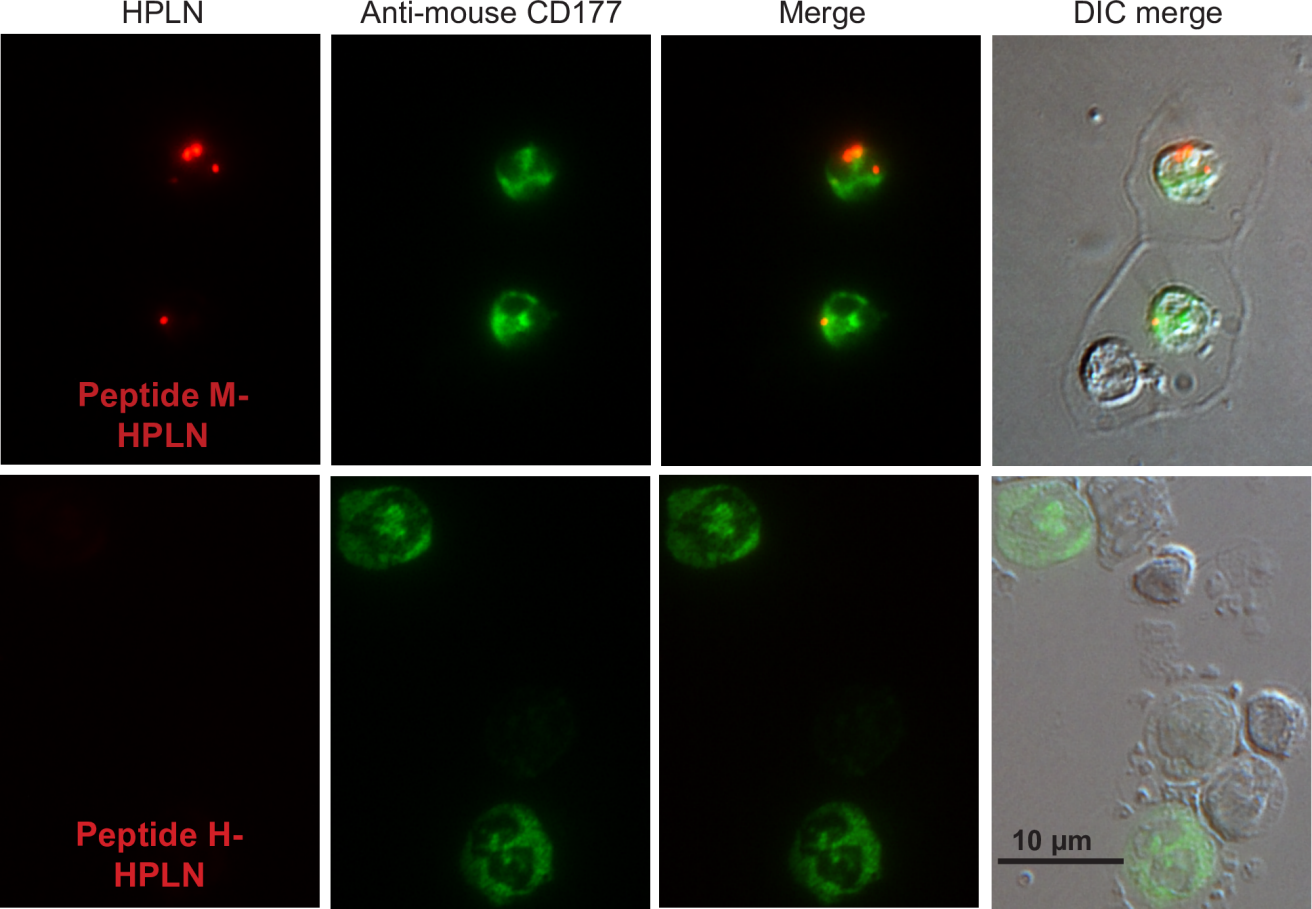
Peptide M-HPLN particles bind CD177-expressing mouse neutrophils in whole blood. Peptide M-HPLN particles were incubated with whole mouse blood for 1 h at 37°C. The white blood cells were collected from the buffy coat and the red blood cells were lysed. The white blood cells were centrifuged onto glass slides, fixed in methanol and stained with rabbit anti-mouse CD177 polyclonal antibody and Alexa Fluor 488 goat anti-rabbit secondary antibody. Peptide H-HPLN particles were used as a negative control. The experiment was carried out twice. The micrographs represent the results from >100 cells. Scale bar, 10 μm.

**Fig 6:**
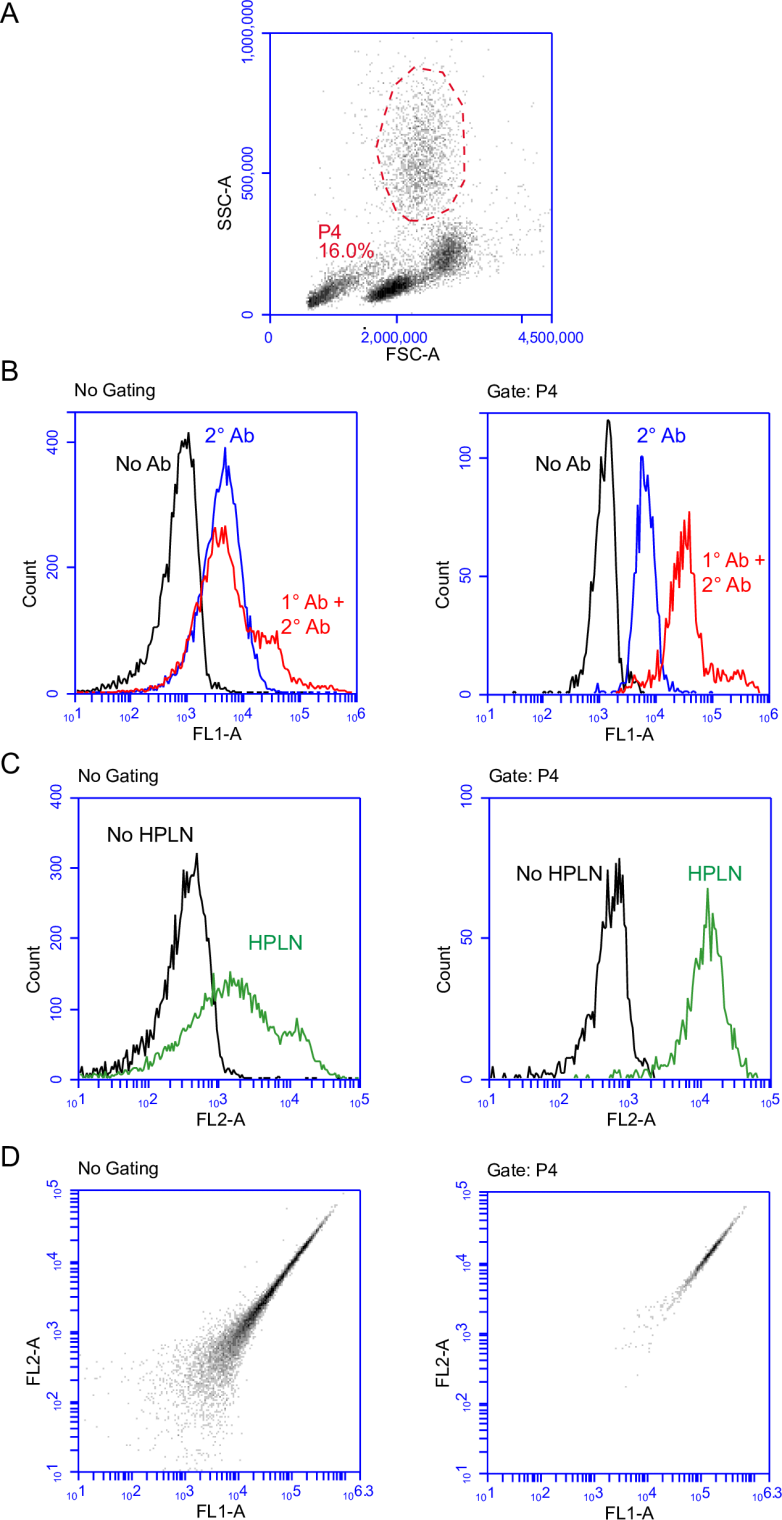
Flow cytometry confirms Peptide H-HPLN binding to CD177-positive neutrophils in a pool of purified human leukocytes. Leukocytes were incubated on ice in the presence or absence of anti-CD177 antibody, washed and incubated on ice in the presence or absence of Peptide H-HPLN and Alexa Fluor 488 goat anti-mouse antibody. **A.** Forward and side scatter plot of human leukocytes (and remaining red blood cells) with the neutrophil population shown in a circle. **B.** FL-1 histogram of CD177 expression. Left panel: Total cell population in the absence and presence of anti-CD177 antibody (1° Ab) and Alexa Fluor 488 goat anti-mouse antibody (2° Ab). Right panel: Gated neutrophil population in the absence or presence of anti-CD177 antibody (1° Ab) and Alexa Fluor 488 goat anti-mouse antibody (2° Ab). **C.** FL2 plot showing Peptide H-HPLN binding. Total cell population in the absence or presence of Peptide H-HPLNs (left panel) and gated neutrophil population in the absence and presence of Peptide H-HPLNs (right panel). **D.** FL1-A and FL2-A plot showing fluorescence of the total cell population (left panel) and gated neutrophil population (right panel). The experiment was carried out twice with similar results. The blood sample shown had a higher percentage of CD177-positive neutrophils than the blood sample from the other donor.

**Fig 7:**
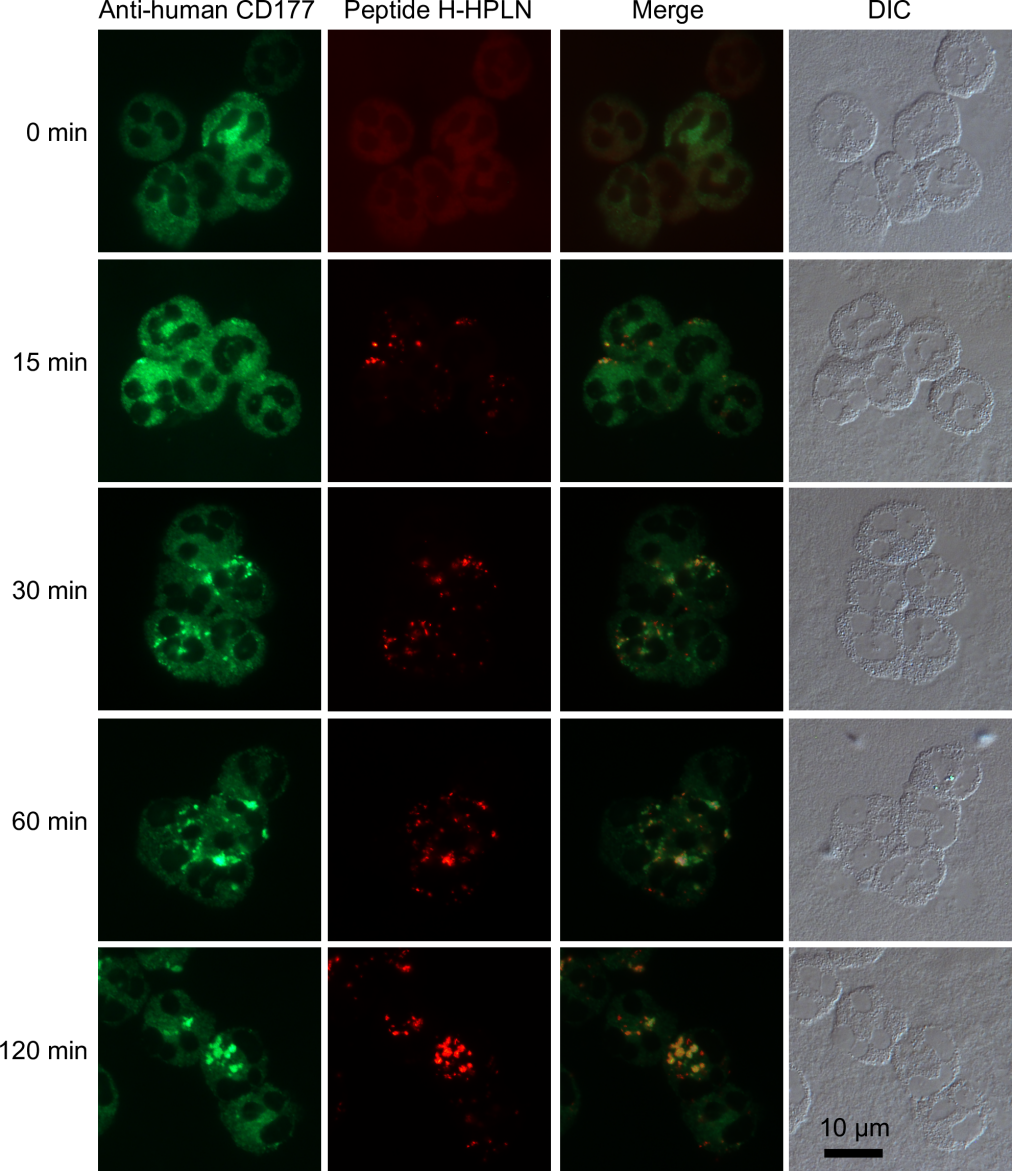
Time course of CD177 and Peptide H-HPLN redistribution in human neutrophils. Purified human neutrophils were incubated with Peptide H-HPLNs at 37°C with aliquots taken at 0, 15, 30, 60 and 120 min. Cells were rinsed with PBS, centrifuged onto glass slides, fixed in methanol and stained with mouse anti-human CD177 antibody and an Alexa Fluor 488 goat anti-mouse secondary antibody. The experiment was carried out once using blood from a donor with a high CD177 expression level. Hundreds of cells were examined and the micrographs are representative of these results. Scale bar, 10 μm.

Therapeutic targeting of neutrophils requires that the binding and internalization of the HPLNs is cell-type specific. We therefore incubated Peptide H-HPLNs with whole human blood to examine the distribution of human CD177 and the HPLNs by immunofluorescence microscopy of white blood cells (after removal of most of the red blood cells). As shown in Fig 4, only cells that stained with anti-human CD177 antibody and had a lobed nucleus—typical of neutrophils—were associated with Peptide H-HPLNs. Similarly, when mouse whole blood was incubated with Peptide M-HPLNs, only cells positive for mouse CD177 showed staining with the Peptide M-HPLN particles. HPLNs containing the human CD177 binding Peptide H did not bind to mouse CD177, suggesting species specificity (Fig 5). The specificity of Peptide H-HPLN was confirmed by flow cytometry—only CD177-positive neutrophils were positive for Peptide H-HPLN (Fig 6). A time course immunofluorescence experiment using purified human neutrophils showed a homogeneous surface distribution of CD177 and Peptide H-HPLN after a 15 min incubation at 37°, whereas incubations for 30, 60 and 120 min showed an increasing amount of redistribution, suggestive of endocytosis (Fig 7). Our attempts to confirm endocytosis using subtilisin failed because of neutrophil sensitivity to the treatment. Incubation with Peptide H-HPLN for 120 min did not show colocalization with the lysosomal marker, LAMP2, in agreement with the results using the CHO transfectants (S1 Fig).

### Knockdown of mouse and human C5aR1 using siRNA and antisense oligonucleotides (ASOs)

**Fig 8:**
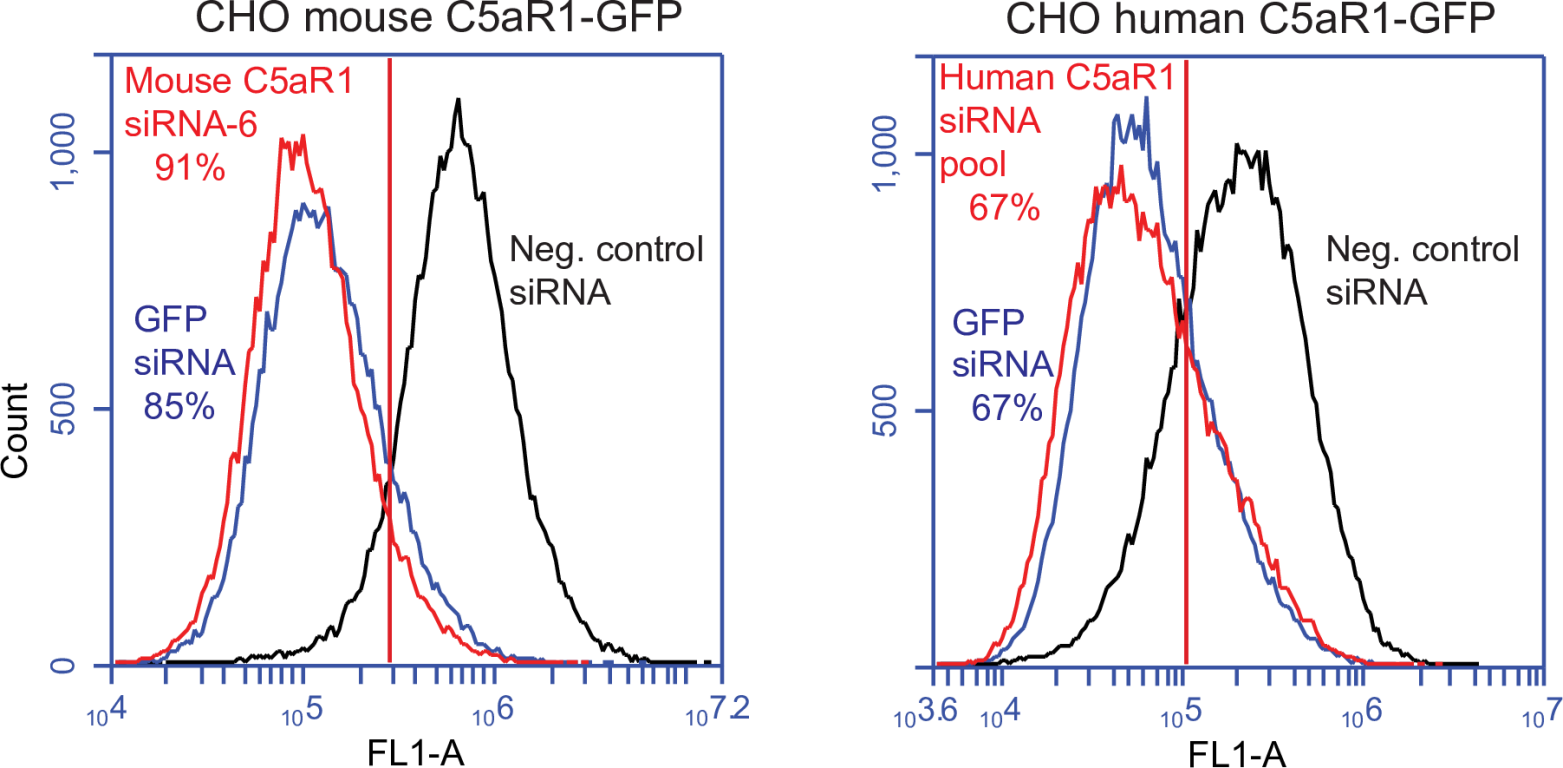
Mouse C5aR1 siRNA and human C5aR1 siRNA pool result in receptor knockdown. CHO cells expressing mouse C5aR1-GFP were transfected with 100 nM mouse C5aR1 ON-TARGETplus SMART siRNA–6, 100 nM GFP siRNA (positive control), or 100 nM negative control siRNA. 72 h post transfection cells were analyzed by flow cytometry to measure the relative expression of mouse C5aR1-GFP (left panel). CHO cells expressing human C5aR1-GFP were transfected with 100 nM human C5aR1 ON-TARGETplus SMARTpool siRNA, 100 nM GFP siRNA (positive control), or 100 nM negative control siRNA (right panel). Relative knockdown is based on the percentage of the cells that are to the left of the gate relative to the negative control sample. The experiment was carried out twice with similar results.

*In vitro* knockdown analysis of human and mouse neutrophils is not possible because of the relatively short half-life of these cells after isolation from blood (~8 hours *in vitro* compared to ~5.4 days *in vivo*) [62]. To examine C5aR1 knockdown, we generated stable cell lines that express various combinations of human or mouse C5aR1, C5aR1-GFP and CD177. Protein knockdown was measured by flow cytometry, and mRNA knockdown was measured by RT-qPCR. For protein knockdown, we used human and mouse C5aR1 with a C-terminal fusion of green fluorescent protein (GFP). The benefits of measuring GFP rather than C5aR1 are three-fold; 1) avoiding any potential background labeling due to non-specific binding of primary and secondary antibodies; 2) avoiding antibody titrations to ensure binding site saturation; 3) providing an internal positive control for knockdown using previously tested and commercially available GFP siRNA [63]. Transfection utilized a variety of proprietary, commercially-available lipid formulations (see Materials and methods), with our ultimate goal being utilization of our targeted HPLNs for this purpose. Our initial experiments using a pool of four different mouse C5aR1 siRNAs showed very poor knockdown compared to our positive GFP siRNA control (S2 Fig). We therefore tested the siRNAs individually and found that only one out of four siRNAs (siRNA-6) resulted in knockdown comparable to GFP siRNA (S3 Fig). This siRNA was used in the subsequent experiments. As shown in Fig 8, the mouse C5aR1 siRNA resulted in a 91% decrease in mouse C5aR1-GFP fluorescence compared to the negative control siRNA, and the GFP siRNA positive control resulted in an 85% decrease. The knockdown of human C5aR1-GFP was somewhat less efficient at 67% for both the human C5aR1 siRNA pool and the GFP siRNA (Fig 8).

**Fig 9:**
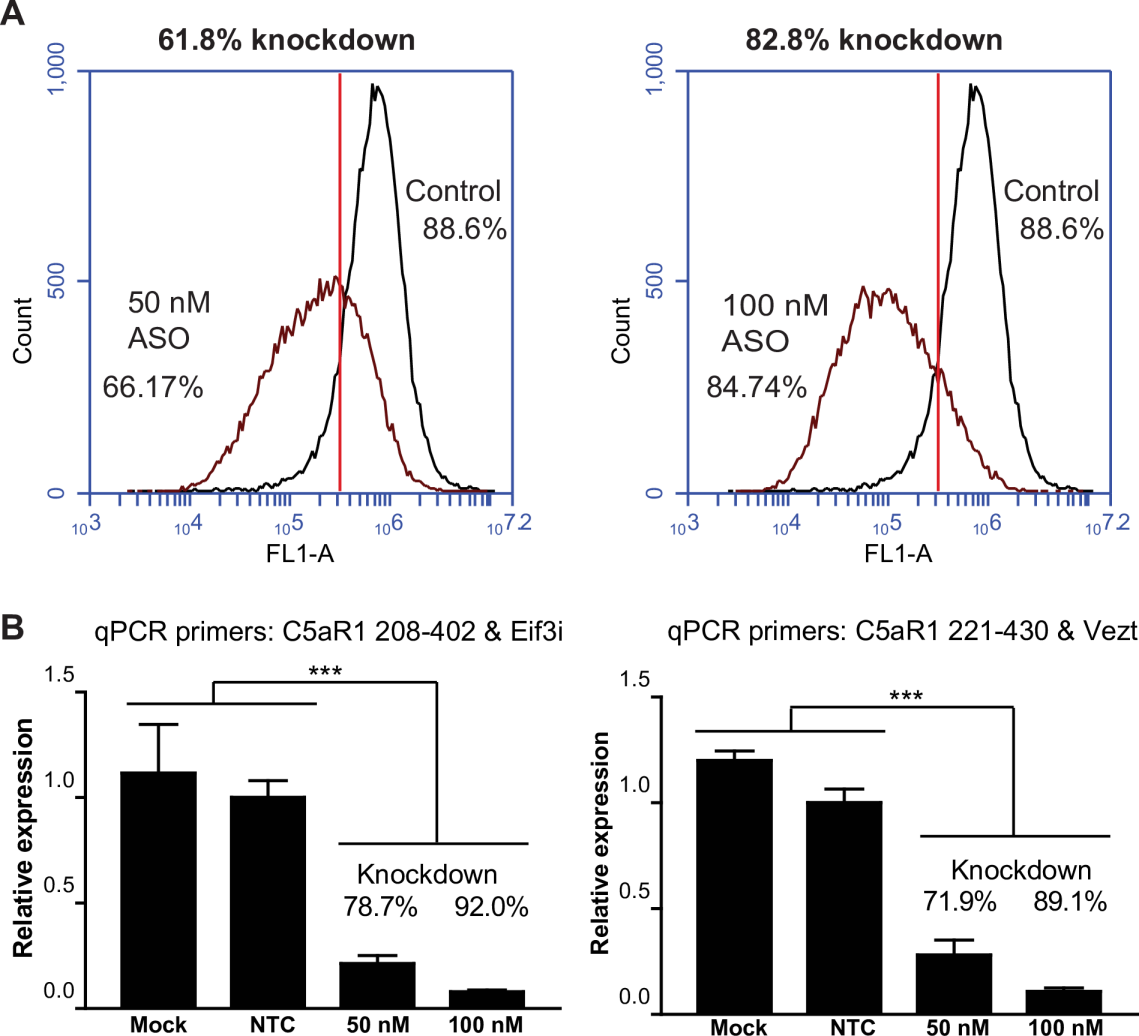
Mouse C5aR1 ASO results in knockdown of mouse C5aR1-GFP in CHO transfectants. CHO cells expressing mouse C5aR1-GFP were transfected with 50 nM or 100 nM LNA GapmeR ASO. The cells were analyzed for C5aR1-GFP expression and mRNA levels 72 h post transfection. **A.** Relative receptor knockdown was measured by flow cytometry. The percentage knockdown was calculated based on the number of cells to the left of the gate relative to the negative control ASO. **B.** Relative gene expression was calculated from quantification cycle (Cq) values obtained by RT-qPCR using the ΔΔCq method. To control for possible experimental variation, the qPCR was carried out using two sets of mouse C5aR1 primers (C5aR1 208–402 and 221–430), and two sets of reference primers. The results in the left panel show the relative quantity of C5aR1 mRNA normalized to Eif3i, and the results in the right panel show the relative quantity of C5aR1 mRNA normalized to Vezt. Mock transfected cells received no ASO and non-targeting control (NTC) cells were transfected with a non-targeting ASO. The RT-qPCR was carried out with triplicate samples ± SD. One-way analysis of variance at 95% confidence interval showed that the relative mRNA expression levels were significantly lower in the ASO treated cells compared to the mock transfected and non-targeting ASO cells (*p* value <0.0001; ***).

As a more stable alternative to siRNA, we also examined knockdown using antisense oligonucleotides from Exiqon. These third generation LNA GapmeR™ ASOs contain a phosphorothioate backbone and locked nucleotides in each end making them far superior to the previous ASOs in potency, specificity, and stability [64, 65]. Our first test compared the knockdown effect of two mouse C5aR1 ASOs. Based on these results (S4 Fig), all future experiments were carried out using ASO1 (sequence shown in List C in S1 File). Flow cytometry 72 h after transfection with ASO1 showed about 85% decrease in the fluorescence of mouse C5aR1-GFP; but with a somewhat larger cell-to-cell variation in receptor knockdown compared to the siRNAs (Fig 9A). To analyze the knockdown further, we carried out RT-qPCR. Reference (housekeeping) genes for RT-qPCR were selected based on their stable expression levels under different conditions of CHO cell growth [48]. These reference genes were also ideal since the mRNA levels were higher (Eif3i) or lower (Vetz) than the mouse C5aR1 mRNA levels, and their primer pairs had been previously been validated (S5 Fig) [46]. We confirmed that the expression levels of the reference genes were not affected by ASO1 transfection (S6 Fig). The results showed about 89–92% knockdown of mouse C5aR1-GFP with 100 nM ASO1, in good agreement with the flow cytometry results (Fig 9B).

### Future research

Cell-specific targeting and cytosolic (or nuclear) delivery of peptides, proteins, and nucleic acids remains a well-known obstacle in drug delivery. In many cases, biotherapeutic agents are sensitive to the low pH and degradative enzymes found in lysosomes [66–68]. To achieve efficient cytosolic delivery of therapeutic cargo that has been loaded into our HPLNs, additional modifications of these nanoparticles may be necessary, and is currently being explored. Future experiments may investigate previously described mechanisms for drug delivery into cells. One strategy is based on a pH-dependent fusogenic peptide (diINF-7) that resembles the N-terminal domain of influenza virus hemagglutinin HA-2 and allows membrane fusion and cytosolic access of endosome-entrapped molecules [69–71]. Another possible approach is the use of a pH-responsive fusogenic peptide, GALA. GALA is a 30 amino acid synthetic peptide that converts from a water-soluble random coil at pH 7.0 to an amphipathic, membrane binding α-helix at pH 5.0 [72]. GALA forms a transmembrane peptide pore comprised of about 10 GALA α-helical monomers arrayed perpendicular to the plane of the membrane, and has been shown to greatly enhance endosomal escape and gene silencing of siRNA-containing nanoparticles [73]. The diINF-7 or GALA fusogenic peptide can be incorporated into the HPLNs in the same way that the targeting peptides are incorporated. Once inside cells, release of the siRNA or ASO cargo is also critical to optimal efficacy. In addition to the fusogenic peptides, the polymer content in the HPLN shell can be optimized to promote opening and nucleotide release [58].

## Conclusions

Neutrophils play an important role not only in innate immune reactions, but also in a wide variety of other functions. Regulation of some of these functions through specific targeting of neutrophils, or a subpopulation of neutrophils, would be therapeutically advantageous. We show here that the CD177-positive population of human and mouse neutrophils can be specifically targeted using HPLNs that display on their surface human and mouse CD177-binding peptides. We also identify siRNAs and ASOs that can be used for knockdown of C5aR1, an important cell surface receptor in migration and activation of neutrophils. Further exploration of these tools may lead to new drugs against disorders as varied as metastatic cancer, heart disease and stroke.

## Supporting information

S1 Fig Peptide H-HPLNs do not colocalize with LAMP2 in human neutrophils. Purified human neutrophils were incubated with Peptide H-HPLNs at 37°C with aliquots taken at 0, 15, 30, 60 and 120 min. Cells were rinsed with PBS, centrifuged onto glass slides, fixed in methanol and stained with mouse anti-human LAMP2 antibody and an Alexa Fluor 488 goat anti-mouse secondary antibody. The experiment was carried out once using blood from a donor with high CD177 expression level. Several hundred neutrophils were examined and the micrographs are representative of these results. Scale bar, 10 μm.
(PDF)

S2 Fig The mouse C5aR1 siRNA SMARTpool resulted in relatively poor receptor knockdown compared to GFP siRNA. CHO cells expressing mouse C5aR1-GFP were transfected with 25–100 nM ON-TARGETplus SMARTpool mouse C5aR1 siRNA or 100 nM GFP siRNA. 72 h post transfection mouse C5aR1-GFP expression was examined by flow cytometry.
(PDF)

S3 Fig Testing of individual mouse C5aR1 siRNAs from the ON-TARGET SMARTpool, identified only one siRNA (-6) with a knockdown efficiency similar to the positive control GFP siRNA. CHO cells expressing mouse C5aR1-GFP were transfected with 100 nM ON-TARGETplus SMARTpool mouse C5aR1 siRNA-5, -6, 7, or 8, or 100 nM GFP siRNA. C5aR1-GFP expression was examined 72 h post transfection by flow cytometry.
(PDF)

S4 Fig Mouse C5aR1 LNA GapmeR ASO1 resulted in somewhat better knockdown of mouse C5aR-GFP than ASO2. CHO cells expressing mouse C5aR1-GFP were mock transfected or transfected with 100 nM ASO1 or 100 nM ASO2. Mouse C5aR1-GFP was measured 72 h after transfection by flow cytometry.
(PDF)

S5 Fig Reference genes for RT-qPCR of CHO transfectans had higher (Eif3i) and lower (Vezt) mRNA copy numbers compared to C5aR1. CHO cells stably expressing mouse C5aR1-GFP were transfected with 50 nM Ms C5aR1_1 LNA GapmeR ASO. 72 h post transfection, RNA was isolated and reverse transcribed. Primers for reference genes, Eifi3 and Vezt, were selected based on previously published work [48]. Primers for mouse C5aR1 were selected using a free online program, PRIMER3, and validated based on MIQE guidelines [50].
(PDF)

S6 Fig Control PCR showed no amplification from total RNA without reverse transcription and no changes in expression levels of Eif3i mRNA after transfection with ASOs. CHO cells stably expressing mouse C5aR1-GFP were transfected with 50 nM or 100 nM Ms C5aR1_1 LNA GapmeR ASO. 72 h post transfection, RNA was isolated and first strand synthesis was carried out in the presence of reverse transcriptase (+RT) or the in the absence of reverse transcriptase (-RT). Melt curve showed a single PCR product, as expected.
(PDF)

S1 File Peptide sequences, siRNA sequences, antisense oligonucleotide sequences, RT-qPCR primer sequences and quantitative PCR cycling parameters. (PDF)

## References

[1] Kloner RA. Does reperfusion injury exist in humans? JAmCollCardioll. 1993;21(2):537–45. 10.1016/0735-1097(93)90700-b 8426022

[2] Buras JA, Rice L, Orlow D, Pavlides S, Reenstra WR, Ceonzo K, et al Inhibition of C5 or absence of C6 protects from sepsis mortality. Immunobiology. 2004;209(8):629–35. doi: 10.1016/j.imbio.2004.09.004 15638131 10.1016/j.imbio.2004.09.004

[3] Guo RF, Riedemann NC, Bernacki KD, Sarma VJ, Laudes IJ, Reuben JS, et al Neutrophil C5a receptor and the outcome in a rat model of sepsis. FASEB Journal. 2003;17:1889–981. doi: 10.1096/fj.03-0009fje 12897064 10.1096/fj.03-0009fje

[4] Jorch SK, Kubes P. An emerging role for neutrophil extracellular traps in noninfectious disease. Nat Med. 2017;23(3):279–87. Epub 2017/03/08. doi: 10.1038/nm.4294 . 28267716 10.1038/nm.4294

[5] Jimenez-Alcazar M, Rangaswamy C, Panda R, Bitterling J, Simsek YJ, Long AT, et al Host DNases prevent vascular occlusion by neutrophil extracellular traps. Science. 2017;358(6367):1202–6. Epub 2017/12/02. doi: 10.1126/science.aam8897 . 29191910 10.1126/science.aam8897

[6] Ward PA. Functions of C5a receptors. J Mol Med (Berl). 2009;87(4):375–8. doi: 10.1007/s00109-009-0442-7 19189071 10.1007/s00109-009-0442-7 PMC2754833

[7] Wright HL, Thomas HB, Moots RJ, Edwards SW. RNA-seq reveals activation of both common and cytokine-specific pathways following neutrophil priming. PLoS One. 2013;8(3):e58598 doi: 10.1371/journal.pone.0058598 PONE-D-12-34202 [pii]. 23554905 10.1371/journal.pone.0058598 PMC3590155

[8] Heller T, Hennecke M, Baumann U, Gessner JE, Vilsendorf AMZ, Baensch M, et al Selection of a C5a receptor antagonist from phage libraries attenuating the inflammatory response in immune complex disease and ischemia reperfusion injury. J Immunol. 1999;163(2):985–94. 10395696

[9] Riley RD, Sato H, Zhao ZQ, Thourani VH, Jordan JE, Fernandez AX, et al Recombinant human complement C5A receptor antagonist reduces infarct size after surgical revascularization. Journal of Thoracic and Cardiovascular Surgery. 2000;120(2):350–8. doi: 10.1067/mtc.2000.107281 10917953 10.1067/mtc.2000.107281

[10] Arumugam TV, Shiels IA, Woodruff TM, Granger DN, Taylor SM. The role of the complement system in ischemia-reperfusion injury. Shock. 2004;21(5):401–9. 15087815 10.1097/00024382-200405000-00002

[11] Proctor LM, Arumugam TV, Shiels I, Reid RC, Fairlie DP, Taylor SM. Comparative anti-inflammatory activities of antagonists to C3a and C5a receptors in a rat model of intestinal ischaemia/reperfusion injury. British Journal of Pharmacology. 2004;142(4):756–64. doi: 10.1038/sj.bjp.0705819 15159277 10.1038/sj.bjp.0705819 PMC1575041

[12] Hoesel LM, Niederbichler AD, Ward PA. Complement-related molecular events in sepsis leading to heart failure. Mol Immunol. 2007;44(1–3):95–102. doi: 10.1016/j.molimm.2006.06.009 16875736 10.1016/j.molimm.2006.06.009

[13] Zhang H, Qin G, Liang G, Li J, Barrington RA, Liu DX. C5aR-mediated myocardial ischemia/reperfusion injury. Biochem Biophys Res Commun. 2007;357(2):446–52. doi: 10.1016/j.bbrc.2007.03.152 17416341 10.1016/j.bbrc.2007.03.152

[14] Rittirsch D, Flierl MA, Nadeau BA, Day DE, Huber-Lang M, Mackay CR, et al Functional roles for C5a receptors in sepsis. Nat Med. 2008;14(5):551–7. doi: 10.1038/nm1753 18454156 10.1038/nm1753 PMC2753858

[15] Weisman HF, Bartow T, Leppo MK, Marsh HC Jr., Carson GR, Concino MF, et al Soluble human complement receptor type 1: in vivo inhibitor of complement suppressing post-ischemic myocardial inflammation and necrosis. Science. 1990;249(4965):146–51. 2371562 10.1126/science.2371562

[16] Niederbichler AD, Hoesel LM, Westfall MV, Gao HW, Ipaktchi KR, Sun L, et al An essential role for complement C5a in the pathogenesis of septic cardiac dysfunction. Journal of Experimental Medicine. 2006;203(1):53–61. doi: 10.1084/jem.20051207 16380509 10.1084/jem.20051207 PMC2118072

[17] Tofukuji M, Stahl GL, Metais C, Tomita M, Agah A, Bianchi C, et al Mesenteric dysfunction after cardiopulmonary bypass: role of complement C5a. Ann Thorac Surg. 2000;69(3):799–807. 10750764 10.1016/s0003-4975(99)01408-3

[18] Arumugam TV, Woodruff TM, Stocks SZ, Proctor LM, Pollitt S, Shiels IA, et al Protective effect of a human C5a receptor antagonist against hepatic ischaemia-reperfusion injury in rats. Journal of Hepatology. 2004;40(6):934–41. doi: 10.1016/j.jhep.2004.02.017 15158333 10.1016/j.jhep.2004.02.017

[19] Hoesel LM, Niederbichler AD, Schaefer J, Ipaktchi KR, Gao H, Rittirsch D, et al C5a-blockade improves burn-induced cardiac dysfunction. J Immunol. 2007;178(12):7902–10. 17548628 10.4049/jimmunol.178.12.7902

[20] Nilupul PM, Ma HK, Arakawa S, Howells DW, Markus R, Rowe CC, et al Inflammation following stroke. J Clin Neurosci. 2006;13(1):1–8. doi: S0967-5868(05)00341-3 [pii]; doi: 10.1016/j.jocn.2005.07.005 16410192 10.1016/j.jocn.2005.07.005

[21] Yamagami S, Tamura M, Hayashi M, Endo N, Tanabe H, Katsuura Y, et al Differential production of MCP-1 and cytokine-induced neutrophil chemoattractant in the ischemic brain after transient focal ischemia in rats. J Leukoc Biol. 1999;65(6):744–9. 10380894 10.1002/jlb.65.6.744

[22] Liu SJ, Zhou SW, Xue CS. Effect of tetrandrine on neutrophilic recruitment response to brain ischemia/reperfusion. Acta Pharmacol Sin. 2001;22(11):971–5. 11749785

[23] Jordan JE, Zhao ZQ, Vinten-Johansen J. The role of neutrophils in myocardial ischemia-reperfusion injury. Cardiovascular Research. 1999;43(4):860–78. 10615413 10.1016/s0008-6363(99)00187-x

[24] Johswich K, Martin M, Bleich A, Kracht M, Dittrich-Breiholz O, Gessner JE, et al Role of the C5a receptor (C5aR) in acute and chronic dextran sulfate-induced models of inflammatory bowel disease. Inflamm Bowel Dis. 2009;15(12):1812–23. doi: 10.1002/ibd.21012 19714742 10.1002/ibd.21012

[25] Boulay F, Naik N, Giannini E, Tardif M, Brouchon L. Phagocyte chemoattractant receptors. Annals of the New York Academy of Sciences. 1997;832:69–84. 9704038 10.1111/j.1749-6632.1997.tb46238.x

[26] Monk PN, Scola AM, Madala P, Fairlie DP. Function, structure and therapeutic potential of complement C5a receptors. Br J Pharmacol. 2007;152(4):429–48. doi: 0707332 [pii]; doi: 10.1038/sj.bjp.0707332 17603557 10.1038/sj.bjp.0707332 PMC2050825

[27] Subramanian H, Kashem SW, Collington SJ, Qu H, Lambris JD, Ali H. PMX-53 as a dual CD88 antagonist and an agonist for Mas-related gene 2 (MrgX2) in human mast cells. Mol Pharmacol. 2011;79(6):1005–13. doi: mol.111.071472 [pii]; doi: 10.1124/mol.111.071472 21441599 10.1124/mol.111.071472 PMC3102546

[28] Schnatbaum K, Locardi E, Scharn D, Richter U, Hawlisch H, Knolle J, et al Peptidomimetic C5a receptor antagonists with hydrophobic substitutions at the C-terminus: increased receptor specificity and in vivo activity. Bioorg Med Chem Lett. 2006;16(19):5088–92. doi: S0960-894X(06)00811-0 [pii]; doi: 10.1016/j.bmcl.2006.07.036 16876401 10.1016/j.bmcl.2006.07.036

[29] Jayne DRW, Bruchfeld AN, Harper L, Schaier M, Venning MC, Hamilton P, et al Randomized Trial of C5a Receptor Inhibitor Avacopan in ANCA-Associated Vasculitis. Journal of the American Society of Nephrology: JASN. 2017;28(9):2756–67. Epub 2017/04/13. doi: 10.1681/ASN.2016111179 ; PubMed Central PMCID: PMCPMC5576933. 28400446 10.1681/ASN.2016111179 PMC5576933

[30] Moriconi A, Cunha TM, Souza GR, Lopes AH, Cunha FQ, Carneiro VL, et al Targeting the minor pocket of C5aR for the rational design of an oral allosteric inhibitor for inflammatory and neuropathic pain relief. Proc Natl Acad Sci U S A. 2014;111(47):16937–42. doi: 1417365111 [pii]; doi: 10.1073/pnas.1417365111 25385614 10.1073/pnas.1417365111 PMC4250151

[31] Kissel K, Santoso S, Hofmann C, Stroncek D, Bux J. Molecular basis of the neutrophil glycoprotein NB1 (CD177) involved in the pathogenesis of immune neutropenias and transfusion reactions. Eur J Immunol. 2001;31(5):1301–9. doi: 10.1002/1521-4141(200105)31:5<1301::AID-IMMU1301>3.0.CO;2-J 11465086 10.1002/1521-4141(200105)31:5<1301::AID-IMMU1301>3.0.CO;2-J

[32] Stroncek DF. Neutrophil-specific antigen HNA-2a, NB1 glycoprotein, and CD177. Curr Opin Hematol. 2007;14(6):688–93. doi: 10.1097/MOH.0b013e3282efed9e [pii]. 17898576 10.1097/MOH.0b013e3282efed9e

[33] Bauer S, Abdgawad M, Gunnarsson L, Segelmark M, Tapper H, Hellmark T. Proteinase 3 and CD177 are expressed on the plasma membrane of the same subset of neutrophils. J Leukoc Biol. 2007;81(2):458–64. doi: jlb.0806514 [pii]; doi: 10.1189/jlb.0806514 17077162 10.1189/jlb.0806514

[34] Sachs UJ, Andrei-Selmer CL, Maniar A, Weiss T, Paddock C, Orlova VV, et al The neutrophil-specific antigen CD177 is a counter-receptor for platelet endothelial cell adhesion molecule-1 (CD31). J Biol Chem. 2007;282(32):23603–12. doi: M701120200 [pii]; doi: 10.1074/jbc.M701120200 17580308 10.1074/jbc.M701120200

[35] Goldschmeding R, van Dalen CM, Faber N, Calafat J, Huizinga TW, van der Schoot CE, et al Further characterization of the NB 1 antigen as a variably expressed 56–62 kD GPI-linked glycoprotein of plasma membranes and specific granules of neutrophils. Br J Haematol. 1992;81(3):336–45. 1382544 10.1111/j.1365-2141.1992.tb08237.x

[36] Bayat B, Werth S, Sachs UJ, Newman DK, Newman PJ, Santoso S. Neutrophil transmigration mediated by the neutrophil-specific antigen CD177 is influenced by the endothelial S536N dimorphism of platelet endothelial cell adhesion molecule-1. J Immunol. 2010;184(7):3889–96. doi: jimmunol.0903136 [pii]; doi: 10.4049/jimmunol.0903136 20194726 10.4049/jimmunol.0903136 PMC4154536

[37] Kuckleburg CJ, Tilkens SB, Santoso S, Newman PJ. Proteinase 3 Contributes to Transendothelial Migration of NB1-Positive Neutrophils. J Immunol. 2012;188(5):2419–26. doi: jimmunol.1102540 [pii]; doi: 10.4049/jimmunol.1102540 22266279 10.4049/jimmunol.1102540 PMC3288489

[38] Bai M, Grieshaber-Bouyer R, Wang J, Schmider AB, Wilson ZS, Zeng L, et al CD177 modulates human neutrophil migration through activation-mediated integrin and chemoreceptor regulation. Blood. 2017;130(19):2092–100. Epub 2017/08/16. doi: 10.1182/blood-2017-03-768507 ; PubMed Central PMCID: PMCPMC5680608. 28807980 10.1182/blood-2017-03-768507 PMC5680608

[39] Gohring K, Wolff J, Doppl W, Schmidt KL, Fenchel K, Pralle H, et al Neutrophil CD177 (NB1 gp, HNA-2a) expression is increased in severe bacterial infections and polycythaemia vera. Br J Haematol. 2004;126(2):252–4. doi: 10.1111/j.1365-2141.2004.05027.x BJH5027 [pii]. 15238147 10.1111/j.1365-2141.2004.05027.x

[40] Stroncek DF, Plachta LB, Herr GP, Dalmasso AP. Analysis of the expression of neutrophil-specific antigen NB1: characterization of neutrophils that react with but are not agglutinated by anti-NB1. Transfusion. 1993;33(8):656–60. Epub 1993/08/01. . 8342232 10.1046/j.1537-2995.1993.33893342747.x

[41] Matsuo K, Lin A, Procter JL, Clement L, Stroncek D. Variations in the expression of granulocyte antigen NB1. Transfusion. 2000;40(6):654–62. Epub 2000/06/24. . 10864984 10.1046/j.1537-2995.2000.40060654.x

[42] Wu Z, Liang R, Ohnesorg T, Cho V, Lam W, Abhayaratna WP, et al Heterogeneity of Human Neutrophil CD177 Expression Results from CD177P1 Pseudogene Conversion. PLoS genetics. 2016;12(5):e1006067 Epub 2016/05/27. doi: 10.1371/journal.pgen.1006067 ; PubMed Central PMCID: PMCPMC4882059. 27227454 10.1371/journal.pgen.1006067 PMC4882059

[43] Suvorova ES, Gripentrog JM, Miettinen HM. Different endocytosis pathways of the C5a receptor and the N-formyl peptide receptor. Traffic. 2005;6:100–15. doi: 10.1111/j.1600-0854.2004.00256.x 15634211 10.1111/j.1600-0854.2004.00256.x

[44] Uthayakumar S, Granger BL. Cell surface accumulation of overexpressed hamster lysosomal membrane glycoproteins. Cell Mol Biol Res. 1995;41:405–20. 8867788

[45] Gripentrog JM, Kantele KP, Jesaitis AJ, Miettinen HM. Experimental evidence for lack of homodimerization of the G protein-coupled human *N*-formyl peptide receptor. Journal of Immunology. 2003;171:3187–93. 10.4049/jimmunol.171.6.3187 12960347

[46] Bruehl RE, Dasgupta F, Katsumoto TR, Tan JH, Bertozzi CR, Spevak W, et al Polymerized liposome assemblies: bifunctional macromolecular selectin inhibitors mimicking physiological selectin ligands. Biochemistry. 2001;40(20):5964–74. Epub 2001/05/16. . 11352731 10.1021/bi002921s

[47] Stewart JC. Colorimetric determination of phospholipids with ammonium ferrothiocyanate. Anal Biochem. 1980;104(1):10–4. Epub 1980/05/01. . 6892980 10.1016/0003-2697(80)90269-9

[48] Bahr SM, Borgschulte T, Kayser KJ, Lin N. Using microarray technology to select housekeeping genes in Chinese hamster ovary cells. Biotechnol Bioeng. 2009;104(5):1041–6. doi: 10.1002/bit.22452 19557832 10.1002/bit.22452

[49] Haimes J, Kelley M. Demonstration of a ΔΔCq calculation method to compute relative gene expression from qPCR data: GE Healthcare UK Limited; 2014 Available from: http://dharmacon.horizondiscovery.com/uploadedfiles/resources/delta-cq-solaris-technote.pdf.

[50] Bustin SA, Benes V, Garson JA, Hellemans J, Huggett J, Kubista M, et al The MIQE guidelines: minimum information for publication of quantitative real-time PCR experiments. Clin Chem. 2009;55(4):611–22. Epub 2009/02/28. doi: 10.1373/clinchem.2008.112797 . 19246619 10.1373/clinchem.2008.112797

[51] Mazzucchelli L, Burritt JB, Jesaitis AJ, Nusrat A, Liang TW, Gewirtz AT, et al Cell-specific peptide binding by human neutrophils. Blood. 1999;93(5):1738–48. 10029604

[52] Jaye DL, Edens HA, Mazzucchelli L, Parkos CA. Novel G protein-coupled responses in leukocytes elicited by a chemotactic bacteriophage displaying a cell type-selective binding peptide. J Immunol. 2001;166(12):7250–9. 11390474 10.4049/jimmunol.166.12.7250

[53] Karathanasis E, Geigerman CM, Parkos CA, Chan L, Bellamkonda RV, Jaye DL. Selective targeting of nanocarriers to neutrophils and monocytes. Ann Biomed Eng. 2009;37(10):1984–92. doi: 10.1007/s10439-009-9702-5 19387833 10.1007/s10439-009-9702-5 PMC4082685

[54] Wang Z, Li J, Cho J, Malik AB. Prevention of vascular inflammation by nanoparticle targeting of adherent neutrophils. Nat Nanotechnol. 2014;9(3):204–10. doi: nnano.2014.17 [pii]; doi: 10.1038/nnano.2014.17 24561355 10.1038/nnano.2014.17 PMC4100792

[55] Chu D, Zhao Q, Yu J, Zhang F, Zhang H, Wang Z. Nanoparticle Targeting of Neutrophils for Improved Cancer Immunotherapy. Adv Healthc Mater. 2016;5(9):1088–93. doi: 10.1002/adhm.201500998 26989887 10.1002/adhm.201500998 PMC4864103

[56] Servid A, Jordan P, O'Neil A, Prevelige P, Douglas T. Location of the bacteriophage P22 coat protein C-terminus provides opportunities for the design of capsid-based materials. Biomacromolecules. 2013;14(9):2989–95. doi: 10.1021/bm400796c 23957641 10.1021/bm400796c PMC3882140

[57] Schwarz B, Madden P, Avera J, Gordon B, Larson K, Miettinen HM, et al Symmetry Controlled, Genetic Presentation of Bioactive Proteins on the P22 Virus-like Particle Using an External Decoration Protein. ACS Nano. 2015;9(9):9134–47. doi: 10.1021/acsnano.5b03360 26266824 10.1021/acsnano.5b03360 PMC4863989

[58] Federman N, Chan J, Nagy JO, Landaw EM, McCabe K, Wu AM, et al Enhanced growth inhibition of osteosarcoma by cytotoxic polymerized liposomal nanoparticles targeting the alcam cell surface receptor. Sarcoma. 2012;2012:126906 doi: 10.1155/2012/126906 23024593 10.1155/2012/126906 PMC3447386

[59] Olmsted J, Strand M. Fluorescence of Polymerized Diacetylene Bilayer Films. J Phys Chem-Us. 1983;87(24):4790–2. PubMed PMID: ISI:A1983RS83200006.

[60] Eckhardt H, Boudreaux DS, Chance RR. Effects of Substituent-Induced Strain on the Electronic-Structure of Polydiacetylenes. J Chem Phys. 1986;85(7):4116–9. PubMed PMID: ISI:A1986E095400050.

[61] Olmsted J, Strand M. Fluorescence of polymerized diacetylene bilayer films. Journal of Physical Chemistry. 1983;87:4790–2.

[62] Pillay J, den Braber I, Vrisekoop N, Kwast LM, de Boer RJ, Borghans JA, et al In vivo labeling with 2H2O reveals a human neutrophil lifespan of 5.4 days. Blood. 2010;116(4):625–7. doi: blood-2010-01-259028 [pii]; doi: 10.1182/blood-2010-01-259028 20410504 10.1182/blood-2010-01-259028

[63] Caplen NJ, Parrish S, Imani F, Fire A, Morgan RA. Specific inhibition of gene expression by small double-stranded RNAs in invertebrate and vertebrate systems. Proc Natl Acad Sci U S A. 2001;98(17):9742–7. Epub 2001/08/02. doi: 10.1073/pnas.171251798 ; PubMed Central PMCID: PMCPMC55523. 11481446 10.1073/pnas.171251798 PMC55523

[64] Chan JH, Lim S, Wong WS. Antisense oligonucleotides: from design to therapeutic application. Clin Exp Pharmacol Physiol. 2006;33(5–6):533–40. doi: CEP [pii]; doi: 10.1111/j.1440-1681.2006.04403.x 16700890 10.1111/j.1440-1681.2006.04403.x

[65] Kauppinen S, Vester B, Wengel J. Locked nucleic acid (LNA): High affinity targeting of RNA for diagnostics and therapeutics. Drug Discov Today Technol. 2005;2(3):287–90. doi: S1740-6749(05)00055-7 [pii]; doi: 10.1016/j.ddtec.2005.08.012 24981949 10.1016/j.ddtec.2005.08.012 PMC7105916

[66] Dominska M, Dykxhoorn DM. Breaking down the barriers: siRNA delivery and endosome escape. J Cell Sci. 2010;123(Pt 8):1183–9. doi: 123/8/1183 [pii]; doi: 10.1242/jcs.066399 20356929 10.1242/jcs.066399

[67] Lonn P, Kacsinta AD, Cui XS, Hamil AS, Kaulich M, Gogoi K, et al Enhancing endosomal escape for intracellular delivery of macromolecular biologic therapeutics. Scientific reports. 2016;6:32301 Epub 2016/09/09. doi: 10.1038/srep32301 ; PubMed Central PMCID: PMCPMC5015074. 27604151 10.1038/srep32301 PMC5015074

[68] Varkouhi AK, Scholte M, Storm G, Haisma HJ. Endosomal escape pathways for delivery of biologicals. Journal of controlled release: official journal of the Controlled Release Society. 2011;151(3):220–8. Epub 2010/11/17. doi: 10.1016/j.jconrel.2010.11.004 . 21078351 10.1016/j.jconrel.2010.11.004

[69] Plank C, Oberhauser B, Mechtler K, Koch C, Wagner E. The influence of endosome-disruptive peptides on gene transfer using synthetic virus-like gene transfer systems. J Biol Chem. 1994;269(17):12918–24. 8175709

[70] Mastrobattista E, Koning GA, van BL, Filipe AC, Jiskoot W, Storm G. Functional characterization of an endosome-disruptive peptide and its application in cytosolic delivery of immunoliposome-entrapped proteins. J Biol Chem. 2002;277(30):27135–43. doi: 10.1074/jbc.M200429200 M200429200 [pii]. 12021269 10.1074/jbc.M200429200

[71] Oliveira S, van R I, Kranenburg O, Storm G, Schiffelers RM. Fusogenic peptides enhance endosomal escape improving siRNA-induced silencing of oncogenes. Int J Pharm. 2007;331(2):211–4. doi: S0378-5173(06)01009-X [pii]; doi: 10.1016/j.ijpharm.2006.11.050 17187949 10.1016/j.ijpharm.2006.11.050

[72] Li W, Nicol F, Szoka FC Jr. GALA: a designed synthetic pH-responsive amphipathic peptide with applications in drug and gene delivery. Adv Drug Deliv Rev. 2004;56(7):967–85. doi: 10.1016/j.addr.2003.10.041 S0169409X03002801 [pii]. 15066755 10.1016/j.addr.2003.10.041

[73] Hatakeyama H, Ito E, Akita H, Oishi M, Nagasaki Y, Futaki S, et al A pH-sensitive fusogenic peptide facilitates endosomal escape and greatly enhances the gene silencing of siRNA-containing nanoparticles in vitro and in vivo. J Control Release. 2009;139(2):127–32. doi: S0168-3659(09)00418-0 [pii]; doi: 10.1016/j.jconrel.2009.06.008 19540888 10.1016/j.jconrel.2009.06.008

