# Supplementary figures and images for: CD177-mediated nanoparticle targeting of human and mouse neutrophils
Source: PLoS One. 2018 Jul 10;13(7):e0200444. doi: 10.1371/journal.pone.0200444 (PMC6039027; doi:10.1371/journal.pone.0200444)

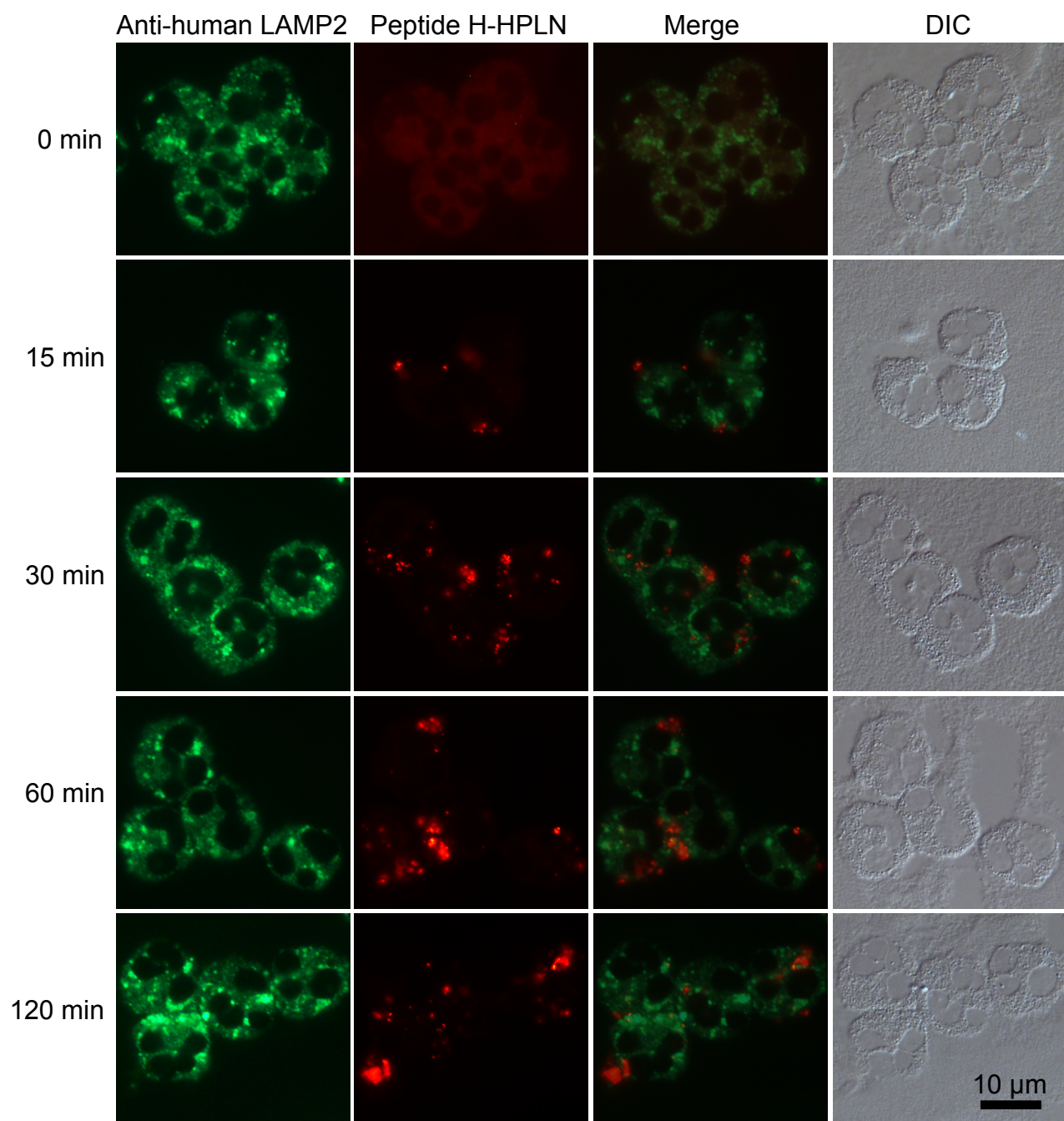

Supplement: S1 Fig — Purified human neutrophils were incubated with Peptide H-HPLNs at 37°C with aliquots taken at 0, 15, 30, 60 and 120 min. Cells were rinsed with PBS, centrifuged onto glass slides, fixed in methanol and stained with mouse anti-human LAMP2 antibody and an Alexa Fluor 488 goat anti-mouse secondary antibody. The experiment was carried out once using blood from a donor with high CD177 expression level. Several hundred neutrophils were examined and the micrographs are representative of these results. Scale bar, 10 μm. (PDF) [file pone.0200444.s001.pdf]

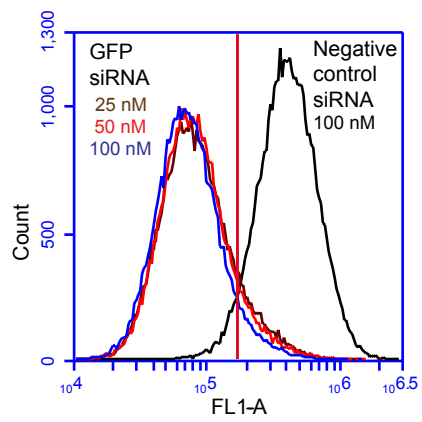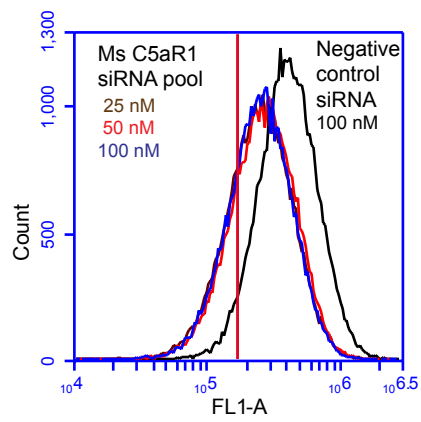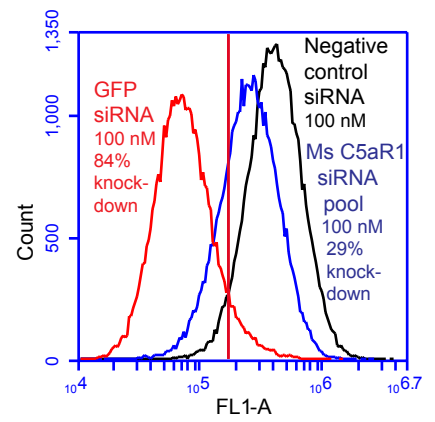

Supplement: S2 Fig — CHO cells expressing mouse C5aR1-GFP were transfected with 25–100 nM ON-TARGETplus SMARTpool mouse C5aR1 siRNA or 100 nM GFP siRNA. 72 h post transfection mouse C5aR1-GFP expression was examined by flow cytometry. (PDF) [file pone.0200444.s002.pdf]

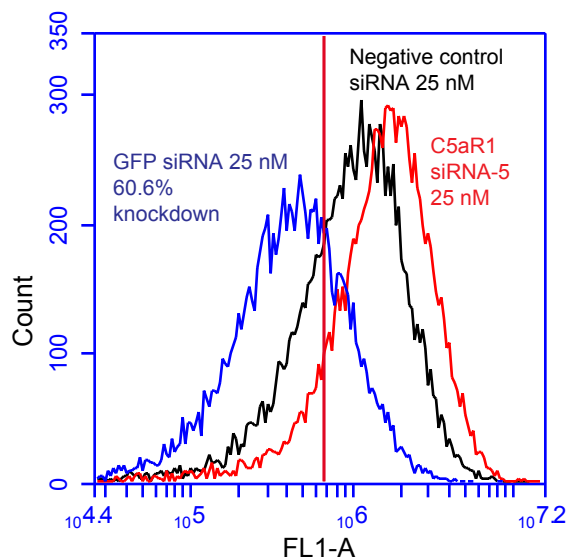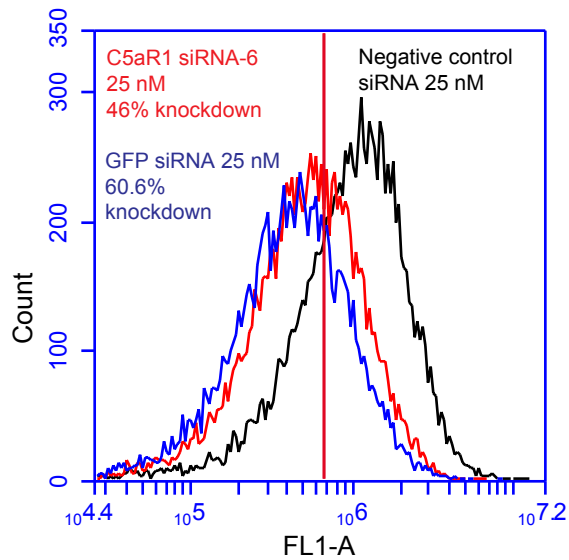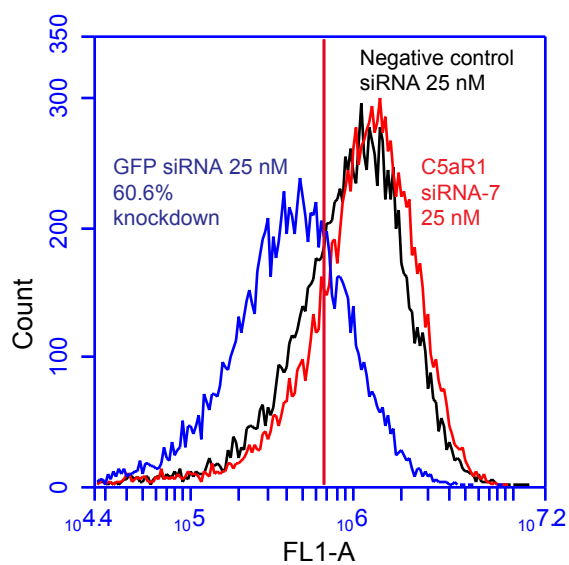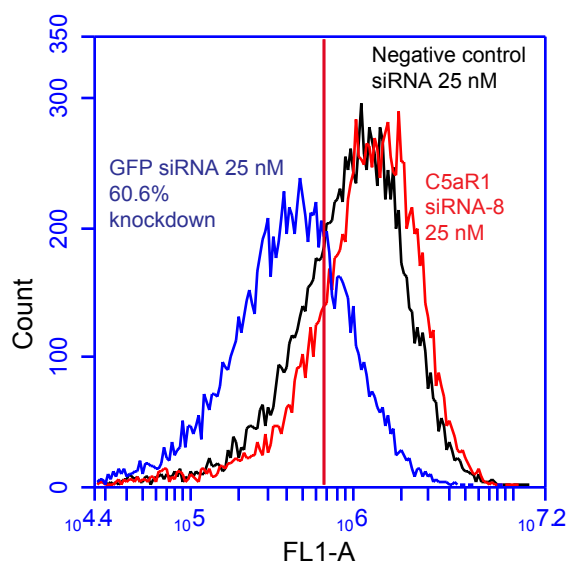

Supplement: S3 Fig — CHO cells expressing mouse C5aR1-GFP were transfected with 100 nM ON-TARGETplus SMARTpool mouse C5aR1 siRNA-5, -6, 7, or 8, or 100 nM GFP siRNA. C5aR1-GFP expression was examined 72 h post transfection by flow cytometry. (PDF) [file pone.0200444.s003.pdf]

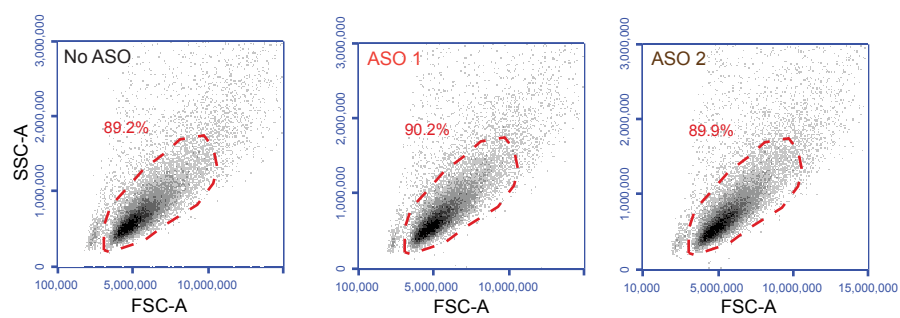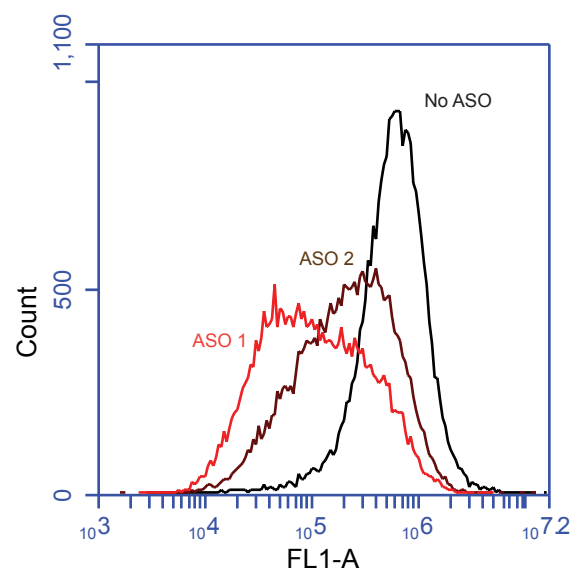

Supplement: S4 Fig — CHO cells expressing mouse C5aR1-GFP were mock transfected or transfected with 100 nM ASO1 or 100 nM ASO2. Mouse C5aR1-GFP was measured 72 h after transfection by flow cytometry. (PDF) [file pone.0200444.s004.pdf]

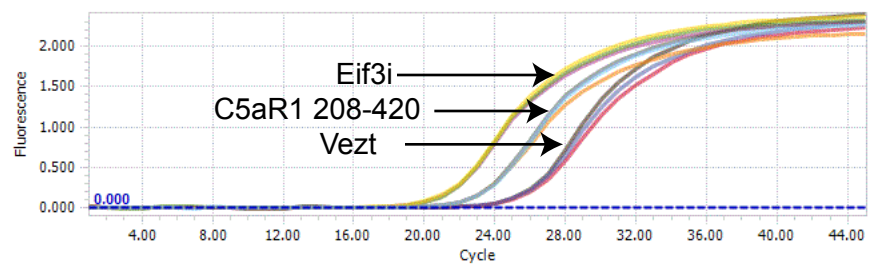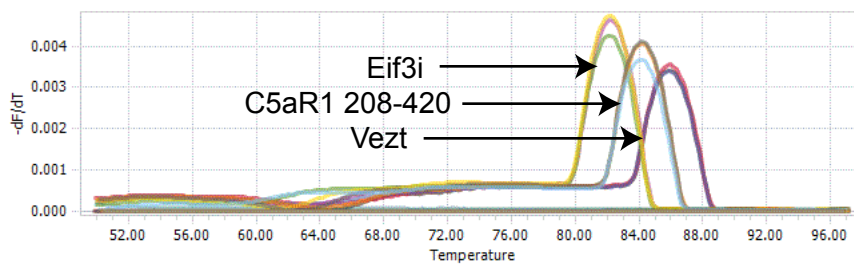

Supplement: S5 Fig — CHO cells stably expressing mouse C5aR1-GFP were transfected with 50 nM Ms C5aR1_1 LNA GapmeR ASO. 72 h post transfection, RNA was isolated and reverse transcribed. Primers for reference genes, Eifi3 and Vezt, were selected based on previously published work [48]. Primers for mouse C5aR1 were selected using a free online program, PRIMER3, and validated based on MIQE guidelines [50]. (PDF) [file pone.0200444.s005.pdf]

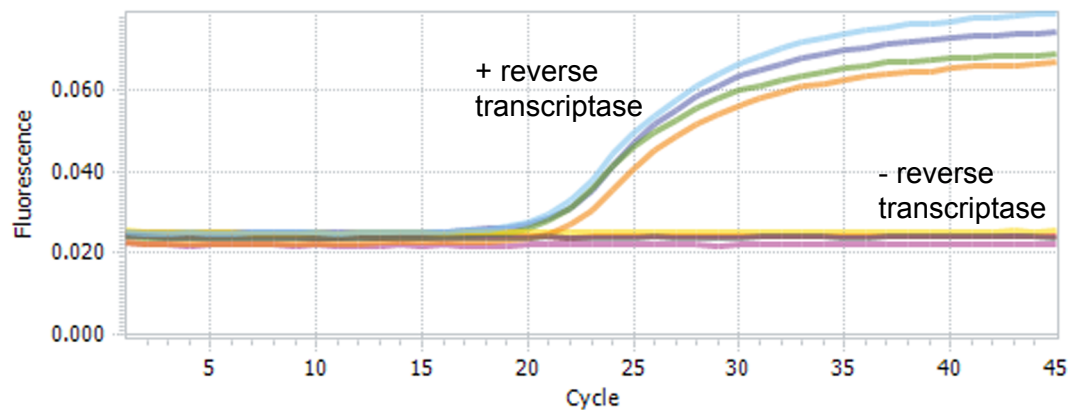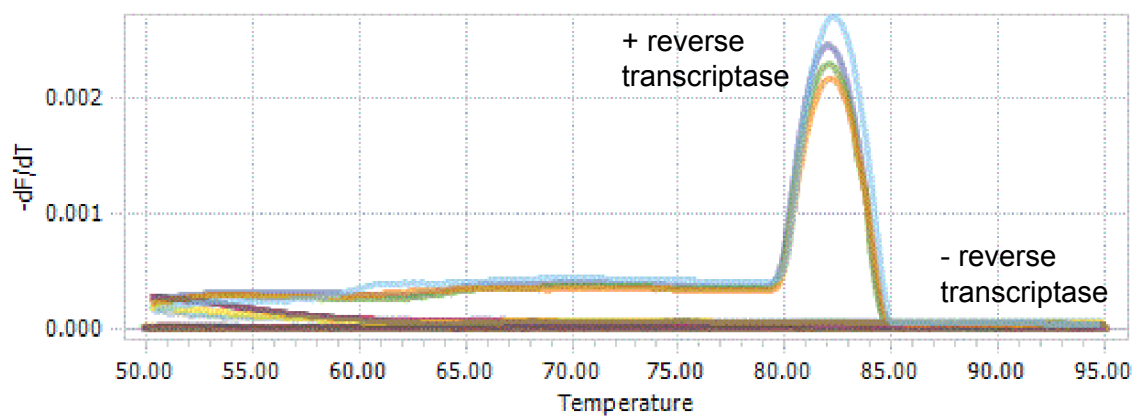

Supplement: S6 Fig — CHO cells stably expressing mouse C5aR1-GFP were transfected with 50 nM or 100 nM Ms C5aR1_1 LNA GapmeR ASO. 72 h post transfection, RNA was isolated and first strand synthesis was carried out in the presence of reverse transcriptase (+RT) or the in the absence of reverse transcriptase (-RT). Melt curve showed a single PCR product, as expected. (PDF) [file pone.0200444.s006.pdf]
