# Supplementary material for: CD177-mediated nanoparticle targeting of human and mouse neutrophils
Source: PLoS One. 2018 Jul 10;13(7):e0200444. doi: 10.1371/journal.pone.0200444 (PMC6039027; doi:10.1371/journal.pone.0200444)
Supplement: S1 File — (PDF) [file pone.0200444.s007.pdf]

Supporting information S1 File

List A. Peptide sequences

Human CD177-binding peptide:	FPLETSHMSAPLGGGC-amide
Scrambled peptide:	SLAMFLTHSPEPGGGC-amide
Mouse CD177-binding peptide:	DFYKPMPNLRITGGGC-amide

List B. siRNA sequences

Dharmacon "ON-TARGET plus siRNA"

Mouse C5aR1 SMART pool

5'-CGUCAACGCCAUCUGGUUU-3'	Catalog # J-043176 -05
5'-GAUAACAGCAGCUUUGAAA-3'	Catalog # J-043176 -06
5'-UACAUCAACUGCUGUGUUA-3'	Catalog # J-043176 -07
5'-GCAGGGAUAGCAAGACUUU-3'	Catalog # J-043176 -08

Human C5aR1 SMART pool

5'-CUACUCACGCUCACGAUUU-3'	Catalog # J-005442-05
5'-GGACUACAGCCACGACAAA-3'	Catalog # J-005442-06
5'-GAGGAGUACUUUCCACCAA-3'	Catalog # J-005442-07
5'-GGAACGUGUUGACUGAAGA-3'	Catalog # J-005442-08

Non-targeting siRNA#1 control

(Proprietary sequence) Catalog # D-001210-01-05

Dharmacon positive control GFP Duplex I siRNA

5'-P GCAAGCUGACCCUGAAGUUCAUGCCGUUCCACUGGGACUUCAAG P-5'

(P = phosphate) Catalog # P-002048-01-20

List C. Antisense oligonucleotide sequences

Mouse C5aR1 LNA™ GapmeR ASOs from Exiqon

ASO-1: 5'-A*A*C*G*G*T*C*G*G*C*A*C*T*A*A*T-3' Order # 580726-1

ASO-2: 5'-A*A*C*G*G*T*C*G*T*G*A*A*C*A*G*G-3' Order # 580726-2

(* position of phosphorothioated DNA bases; the location of locked nucleic acids is proprietary)

Control non-targeting ASO from Integrated DNA Technologies

5'-mC*mA*mU*mC*mA*C*C*A*C*T*T*T*G*A*G*mC*mG*mU*mU-3'

(m: 2′-O-Methyl bases; * position of phosphorothioated DNA bases)

List D. RT-qPCR primer sequences

Mouse C5aR1 nucleotides 208-402 (195 bp)

Forward primer: 5'-GTCACCGCCATCTGGTTTCT-3'

Reverse primer: 5'-ACGGTCGGCACTAATGGTAG-3'

Mouse C5aR1 nucleotides 221-430 (210 bp)

Forward primer: 5'-GGTTTCTGAATCTGGCGGTG-3'

Reverse primer: 5'-ACCAGATGGGCTTGAACACC-3'

Control genes:

CHO Eif3i (166 bp)

Forward primer: 5'-CCACAACTTCCACCAGGATT-3'

Reverse primer: 5'-ATGCGGACGTAACCATCTTC-3'

CHO Vezt (200 bp)

Forward primer: 5'-GTGTGAAAGTGGGGCTGAAT-3'

Reverse primer: 5'-GTTCCTGCATGGTGGTGAAT-3'

Table A. Quantitative PCR cycling parameters

**Conditions**	**Number of cycles**	**Ramp**	**Duration**	**Target**
Preincubation	1	4.0ºC/s	900 s	95ºC
Amplification	45	2.2ºC/s	15 s	94ºC
		2.2ºC/s	15 s	55ºC
		4.0ºC/s	15 s	70ºC
Melting	1	4.4ºC/s	10 s	95ºC
		2.2ºC/s	60 s	50ºC
		-	-	95ºC
Acquisition Mode: Continuous, 5 readings/ºC				
Cooling	1	2.2ºC	60s	37ºC
